# Temporomandibular joint atlas for detection and grading of juvenile idiopathic arthritis involvement by magnetic resonance imaging

**DOI:** 10.1007/s00247-017-4000-0

**Published:** 2017-11-13

**Authors:** Christian J. Kellenberger, Thitiporn Junhasavasdikul, Mirkamal Tolend, Andrea S. Doria

**Affiliations:** 1 Department of Diagnostic Imaging, University Children’s Hospital Zürich, Zürich, Switzerland; 2 Children’s Research Centre, University Children’s Hospital Zürich, Zürich, Switzerland; 3 Department of Diagnostic and Therapeutic Radiology, Faculty of Medicine Ramathibodi Hospital, Mahidol University, Bangkok, Thailand; 4 Department of Diagnostic Imaging, The Hospital for Sick Children, Toronto, Canada; 5 Institute of Medical Science, University of Toronto, Toronto, Canada; 6 Department of Medical Imaging, University of Toronto, Toronto, Canada

**Keywords:** Children, Juvenile idiopathic arthritis, Magnetic resonance imaging, Synovitis, Temporomandibular joint

## Abstract

Contrast-enhanced magnetic resonance imaging (MRI) is considered the diagnostic standard for identifying involvement of the temporomandibular joint by juvenile idiopathic arthritis. Early or active arthritis is shown as bone marrow oedema, joint effusion, synovial thickening and increased joint enhancement. Subsequent joint damage includes characteristic deformity of the mandibular condyle, bone erosion, disk abnormalities and short mandibular ramus due to impaired growth. In this pictorial essay, we illustrate normal MRI findings and growth-related changes of the temporomandibular joint in children. The rationale and practical application of semiquantitative MRI assessment of joint inflammation and damage are discussed and presented. This atlas can serve as a reference for grading temporomandibular joint arthritis according to the scoring systems proposed by working groups of OMERACT (Outcome Measures in Rheumatology and Clinical Trials) and the EuroTMjoint research network. Systematic assessment of the level of inflammation, degree of osteochondral deformation, and growth of the mandibular ramus by MRI may aid in monitoring the course of temporomandibular joint arthritis and evaluating treatment options.

**Electronic supplementary material:**

The online version of this article (10.1007/s00247-017-4000-0) contains supplementary material, which is available to authorized users.

## Introduction

Magnetic resonance imaging (MRI) has become the standard for assessing the temporomandibular joint in children with juvenile idiopathic arthritis because both joint inflammation and joint damage can be evaluated [1, 2]. Contrast-enhanced MRI seems to remain the only method for reliably detecting early arthritis of the temporomandibular joint [3–7] and may directly impact treatment decisions [8, 9]. For assessing and monitoring the course of arthritis, grading of the MRI findings is essential [10–13]. Evaluating the level of inflammation, the degree and course of osteochondral deformity, as well as measuring the growth of the mandibular ramus in the long term is needed for assessing the effect of systemic or specific treatments targeting the temporomandibular joint [14]. While MRI without gadolinium-based contrast readily shows osseous changes and advanced inflammation, contrast application is indispensable for detecting early synovitis and assessing the degree and course of the inflammatory activity.

In this pictorial essay, we discuss and illustrate the normal MRI appearance as well as age- and growth-dependent changes of the temporomandibular joint in children. The technique and rationale for semiquantitative grading of temporomandibular joint inflammation and damage are presented. The illustrations in the article and in a supplemental collection of figures (Online Resource 1) are intended to serve as references for both the additive score proposed by the OMERACT (Outcome Measures in Rheumatoid Arthritis and Clinical Trials) MRI in juvenile idiopathic arthritis working group [15] and the progressive score proposed by members of the EuroTMjoint research network [2].

## Normal temporomandibular joint morphology and MRI appearance in children

The bilateral temporomandibular joints are ginglymoarthrodial joints at the skull base enabling the complex motion of the jaw necessary for mastication and speech [16]. Between the upper temporal bone and the lower mandibular condyle, the joint is divided into two separate synovial compartments by the biconcave articular disk, which is a fibrocartilaginous extension of the joint capsule. The lower joint compartment allows for rotational motion and the upper compartment for anterior translation of the condyle during mouth opening. While in the peripheral joint recesses the joint capsule is lined by synovial membrane, the fibrocartilaginous surfaces of the temporal bone and mandibular condyle as well as the articular disk are normally avascular and void of synovium [17].

### Osseous components

**Fig. 1:**
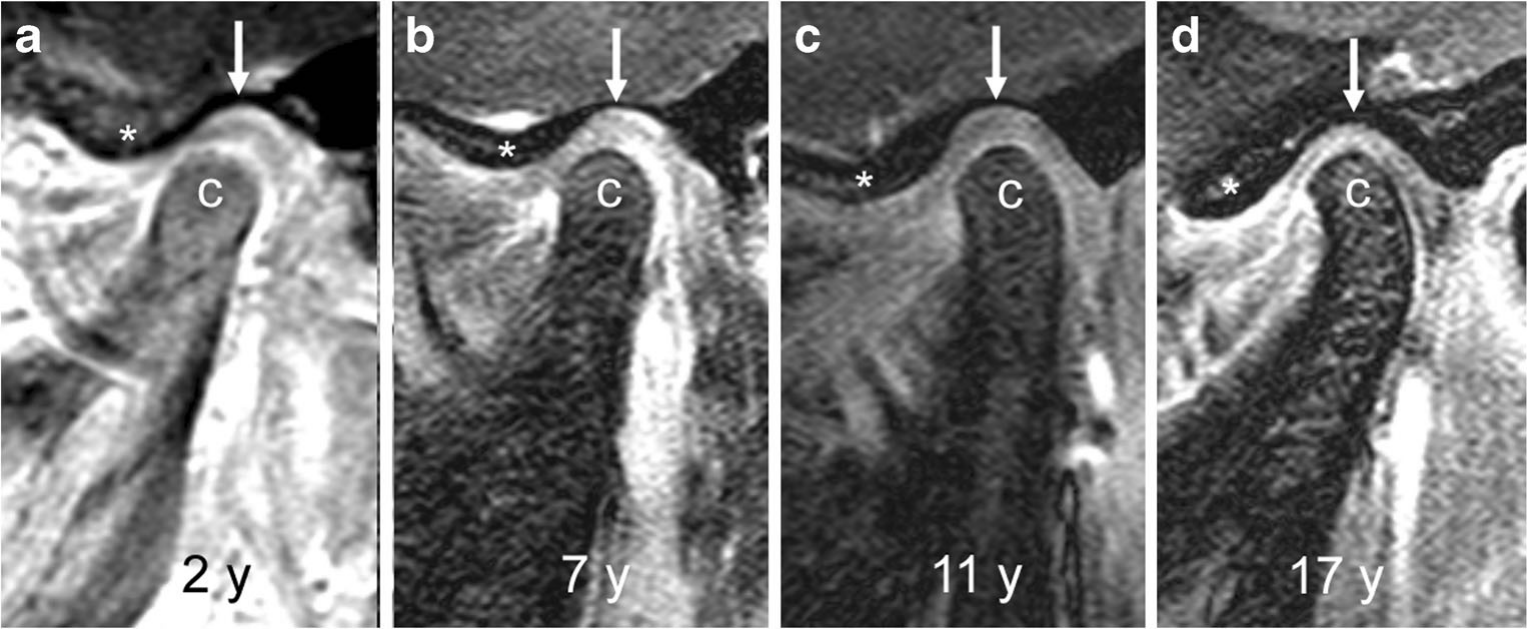
Age-dependent bony configuration of the temporomandibular joint. **a**-**d** Sagittal oblique gradient echo images (TR/TE 10/4.2 ms, flip angle 20°) from different children, obtained, at ages 2 years (**a**), 7 years (**b**), 11 years (**c**) and 17 years (**d**) show changing configurations of the mandibular condyle (c). In the 2-year-old child (a), the superior articular contour is round with a straight condylar neck. With increasing age and growth (**b**-**d**), the condylar neck gains an anterior tilt and the head appears more angular with less rounding of the anterior-superior joint surface. Articular eminence (*) height increases and glenoid fossa (arrows) gets deeper with increasing age

Normally, the temporal and mandibular joint surfaces are delineated by a smooth continuous line representing subchondral bone (Fig. 1), which is most conspicuous on gradient echo images due to the good contrast between mineralised bone with low signal intensity and surrounding soft tissues with high signal intensity [18]. The temporal articular component entails the posterior glenoid or mandibular fossa and the anterior articular eminence resulting in an s-shaped configuration on sagittal oblique images. In young children, the temporal joint surface is rather flat with shallow mandibular fossa [19, 20]. With further growth, the mandibular fossa deepens and the articular eminence gains in height gradually with the adult shape reached around puberty. The mandibular articular component is also subject to growth-related changes in configuration. In the axial plane, the shape of the condyle changes from round to oval due to the increasing ratio between lateral and anterior-posterior dimensions [21]. On sagittal oblique images in a young child up to 5 years of age, the superior contour of the condylar head is round with a straight condylar neck. With increasing age and growth, the condylar neck gains an anterior tilt and the condylar head appears with a more angular shape, with less rounding of the anterior-superior joint surface (Fig. 1) [21, 22]. In the coronal plane, the convex superior joint surface becomes flatter with growth.

**Fig. 2:**
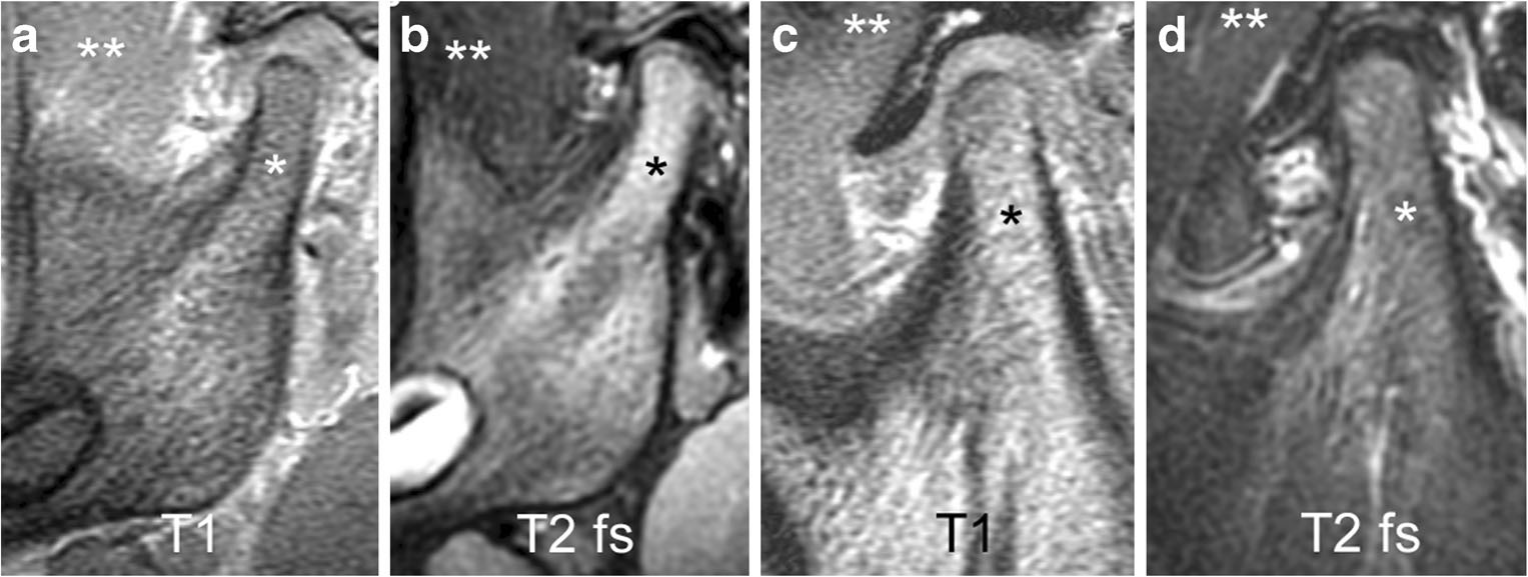
Mandibular bone marrow signal. **a**-**b** Sagittal oblique images from a 2-year-old boy with predominantly red bone marrow in the mandible. Haematopoietic marrow (* ) shows low signal intensity on T1-weighted gradient echo image without fat saturation (TR/TE 300/4.2 ms, flip angle 80°, **a**), which is iso- or hypointense compared to muscle (**), and intermediate signal intensity on fluid-sensitive fast spin echo image with spectral fat saturation (TR/TE 5,400/77 ms), **b**), which is hyperintense compared to muscle (**). **c**-**d** Sagittal oblique images in a 12-year-old boy with predominantly yellow bone marrow. Fatty marrow (*) shows high signal intensity on T1-weighted gradient echo image without fat saturation (TR/TE 300/4.2 ms, flip angle 80°, **c**), which is hyperintense compared to muscle (**), and intermediate to low signal intensity on fluid-sensitive fast spin echo image with spectral fat saturation (TR/TE 5,400/77 ms, **d**), which is almost isointense to muscle (**). Note the normal shape of the articular disk resembling a bow tie with low signal intensity on the fluid-sensitive fast spin echo images. T1 T1-weighted gradient echo, T2 fs fat saturated T2-weighted

Bone marrow composition in the mandible changes during growth, from predominantly haematopoietic marrow initially to mostly fatty marrow later on (Fig. 2) [22, 23]. On T1-weighted and fluid-sensitive images (T2-weighted with fat saturation or short tau inversion recovery [STIR]), the signal intensity of the temporal and mandibular bones reflect the proportions of haematopoietic and fatty marrow. In an infant, bone marrow signal intensity is low on T1-weighted images (isointense to muscle) and intermediate on fluid-sensitive sequences (hyperintense to muscle and hypointense to fluid). With older age and the increasing proportion of fatty marrow, signal intensity eventually becomes the same as subcutaneous fatty tissue on all sequences.

### Articular disk

**Fig. 3:**
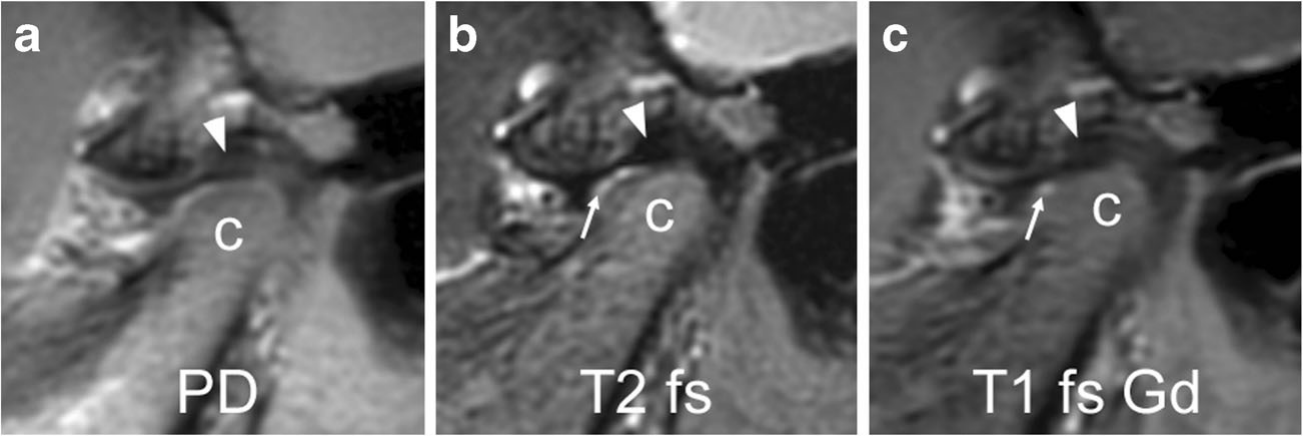
Normal articular disk in a 6-year-old girl. **a**-**c** Proton density weighted (TR/TE 3,000/13 ms, **a**), fat-saturated T2-weighted (TR/TE 5,400/77 ms, **b**), and postcontrast fat-saturated T1-weighted (TR/TE 670/10 ms, **c**) fast spin echo images all show a normal biconcave shape of the articular disk and the posterior band (arrowheads) located at the 11 o’clock position of the mandibular condyle (c). Note normal joint enhancement (arrow in **c**) confined to small amounts of fluid in the anterior lower joint space (arrow in **b**) and mild flattening of the anterior portion of condyle. PD proton density, T1 fs Gd contrast-enhanced fat saturated T1-weighted, T2 fs fat saturated T2-weighted

The normal articular disk is well visualised on fast spin echo images as low signal intensity fibrocartilaginous biconcave structure interposed between the temporal bone and the mandibular condyle (Figs. 2 and 3) [2]. On sagittal oblique images, the disk resembles a bow tie with the anterior and posterior triangular bands joined by a thinner central intermediate zone. The posterior band of the disk is normally located at an 11 to 12 o’clock position in relation to the condyle with the mouth closed, whereas with the mouth opening both the condyle and disk translate anteriorly beneath the articular eminence, so that the central disk portion overlies the condylar apex [24].

### Joint fluid

**Fig. 4:**
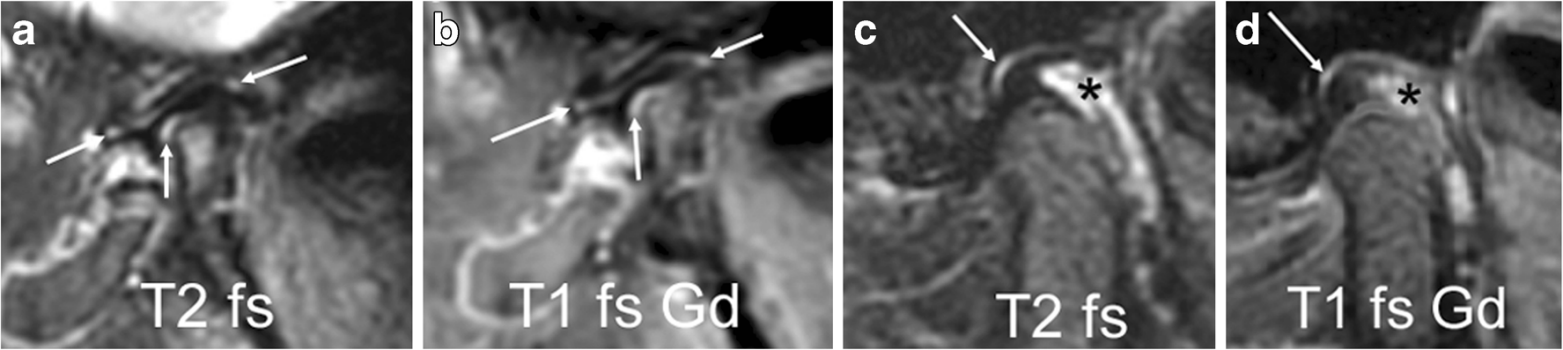
Normal joint fluid and enhancement. **a**-**b** Sagittal oblique images from a 3-year-old girl show normal amounts of fluid in joint recesses. Fluid-sensitive image (TR/TE 5,400/77 ms, fat-saturated, **a**) shows high signal intensity (arrows). On contrast enhanced fat saturated T1-weighted image (TR/TE 670/10 ms, **b**) there is corresponding enhancement (arrows). **c**-**d** Sagittal oblique fast spin echo images from a 13-year-old girl show normal amounts of fluid (arrow) in the joint recesses, with high signal intensity on fluid-sensitive images (**c**) and enhancement following contrast application shown as high signal intensity on post-contrast fat-saturated T1-weighted images (**d**). The slightly expanded venous plexus in the retrodiskal tissue shows high signal intensity on T2-weighted images (* in **c**) and enhancement (* in **d**). T1 fs Gd contrast-enhanced fat saturated T1-weighted, T2 fs fat saturated T2-weighted

As with any other synovial joint, the temporomandibular joint contains small amounts of joint fluid derived from plasma by dialysis and secreted by the synovial membrane [16]. Visibility of synovial fluid on MRI depends on its amount as well as on the orientation, spatial resolution and type of sequence used [23]. While a normal physiological amount of joint fluid is not apparent on T1-weighted images, it is readily detected on T2-weighted images as an area with high signal intensity similar to that of other fluid (i.e. isointense signal compared to cerebrospinal fluid) [25]. Small dots or lines of high signal intensity within the joint recesses, not exceeding 1 mm width, can be considered a physiological amount of joint fluid (Figs. 3 and 4) and should not be interpreted as joint effusion [23].

### Contrast enhancement

**Fig. 5:**
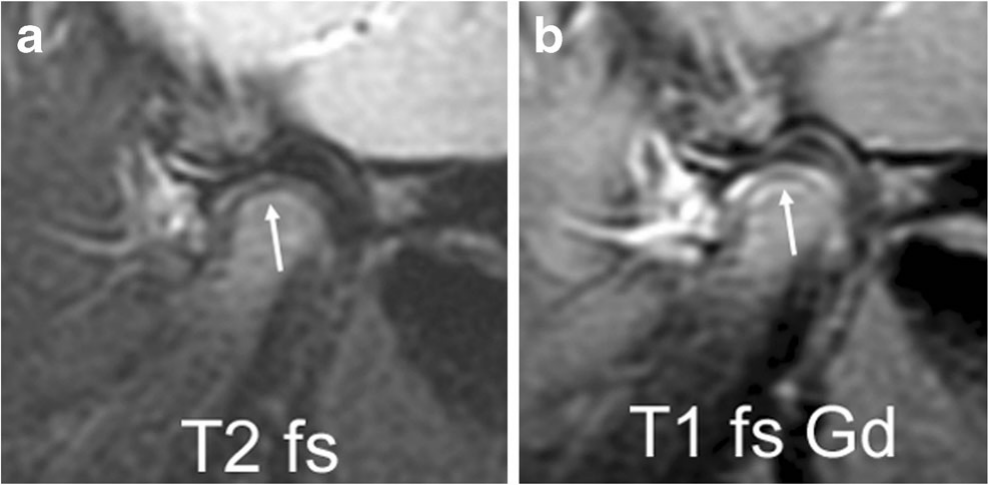
Mandibular growth zone. Sagittal oblique fast spin echo images in a 3-year-old girl show the mandibular growth zone at the surface of the condyle. **a** Beneath the low signal intensity line of cartilage and subchondral bone, this is seen as a narrow zone of high signal intensity (arrow) on fluid-sensitive sequence (TR/TE 5,400/77 ms, fat-saturated). **b** On postcontrast fat-saturated T1-weighted images (TR/TE 670/10 ms) there is corresponding enhancement. These findings represent the well vascularised zone of endochondral ossification. Note enhancement of the whole lower joint compartment as well as anterior recess of upper compartment. T1 fs Gd contrast-enhanced fat saturated T1-weighted, T2 fs fat saturated T2-weighted

Following intravenous application of gadolinium-based contrast agents, all vascularised tissues show an increase of signal intensity on T1-weighted images. Such contrast enhancement has been demonstrated in the bone marrow and joint compartments of normal temporomandibular joints in children by the signal-to-noise ratio increase from unenhanced to postcontrast T1-weighted images [23, 26], as well as by dynamic contrast-enhanced gradient echo imaging [27]. The joint compartment, consisting of the synovial membrane and small amounts of fluid, shows strong enhancement within the first 2 min and a further steady increase beyond 6 min after contrast administration. While signal intensity of vascularised tissues (veins, muscle and synovium) decreases again within minutes, as the intravascular gadolinium concentration falls, diffusion of the contrast agent into the synovial fluid continues and the high signal intensity of fluid on T1-weighted images is retained for at least 1 h [28]. Because normal amounts of joint fluid show high signal intensity (isointense to veins) on fat-saturated T1-weighted images obtained directly after contrast application, we assume that contrast diffusion into the small joint is almost immediate [23]. Initially, any T1-weighted image shows enhancement within areas of normal amounts of fluid in joint recesses as delineated on corresponding fluid-sensitive images (Figs. 3 and 4) [23]. Intra-articular areas without detectable joint fluid maintain signal intensity similar to that of muscle on early images, but may increasingly enhance on later images with high signal intensity seen within the entire joint space including portions void of synovium. Enhancement of bone marrow is evidenced by its signal intensity following that of enhancing muscle on fat-saturated T1-weighted images. The growth zone of the mandibular condyle may show more enhancement than the adjacent bone marrow, resulting in a thin subchondral hyperintense line at the surface of the mandibular head (Fig. 5). Another enhancing structure of the temporomandibular joint is the retrodiskal venous plexus, which distends when the condyle is in an anterior position in relation to the mandibular fossa and becomes visible as a hyperintense structure on T2-weighted or contrast-enhanced T1-weighted images (Fig. 4). Such enhancing retrodiskal tissue (bilaminar zone) should not be mistaken for enhancing synovium or pannus.

## Manifestations of temporomandibular joint arthritis (Figs. 3, 5, 6, 7, 8, 9, 10, 11, 12, 13, 14, 15, 16, 17, 18, 19, 20, 21, 22, 23 and 24)

Early inflammation of the synovial membrane is histologically characterised by synovial hypertrophy with cellular infiltrates, oedema, and increased vascularity [29]. These pathological features of temporomandibular joint arthritis are evident on MRI as joint effusion, synovial thickening, bone marrow oedema and increased joint enhancement [2, 6, 26, 30]. Prolonged inflammation may lead to disturbance of joint formation and damage of the osteochondral structures. With the main growth zones of the mandible located within the joints at the surface of the condyles, covered only by a thin layer of fibrocartilage, arthritis of the temporomandibular joint may lead to growth impairment of the mandible with shortening of the ramus resulting in micrognathia, retrognathia or mandibular asymmetry [1]. Definition, terminology and classification of MRI manifestations have been inconsistent and variable between previously reported scoring systems [11, 31, 32]. For example the term pannus, which is histologically defined as inflammatory tissue that may invade the joint space, cartilage or bone, has been used variably on MRI. Any perceptible thickening of the synovial membrane could be considered pannus. Nevertheless, some have used the term to describe inflammatory tissue expanding the joint space [11], while others have reserved the term for non-enhancing soft tissue in the joint compartments [31]. The following item definitions, grading and division of MRI signs in an inflammatory domain and damage domain have been reached by consensus among experts of the OMERACT and EuroTMjoint research network working groups [2, 15]. The scoring system proposed by the OMERACT working group is semiquantitative and additive, where the total score is the sum of the individually graded items (Table 1) [15, 33]. The scoring system used by members of the EuroTMjoint research network is progressive, where inflammation and deformation grades are determined by the presence of the defined items (Table 2) [2, 11].

**Table 1:** Additive scoring system for assessing inflammation and damage of temporomandibular joint by magnetic resonance imaging

Item	Definition	Grade 0	Grade 1	Grade 2
A) Inflammatory domain (possible total scores 0–8)				
Bone marrow oedema	Compared to the mandibular ramus, hyperintense marrow signalling within the condyle on fluid-sensitive images, and/or hypointense signalling on precontrast T1-weighted images without fat saturation.	Absent	Present	
Bone marrow enhancement	Compared to the mandibular ramus, hyperintense marrow signalling within the condyle on postcontrast T1-weighted fat-saturated images.	Absent	Present	
Joint effusion	Increased joint fluid with isointense signalling of joint space compared to that of cerebrospinal fluid on fluid-sensitive images.	Absent ≤1 mm fluid in joint recess.	Small >“>1 and ≤2 mm thickness in recess or involving entire joint compartment.	Large >2 mm thickness in recess or involving entire joint compartment.
Synovial thickening	Thickened synovial lining of the joint compartments with intermediate signal intensity on fluid-sensitive images.	Absent No synovium visible (apparent joint compartment ≤1 mm width).	Mild >1 and ≤2 mm thickness at the point of maximum synovial thickening.	Moderate/severe >2 mm thickness at the point of maximum synovial thickening.
Joint enhancement	Signal intensity of the synovium, capsule and joint fluid higher than that of muscle on postcontrast T1-weighted fat-saturated images.	Normal High signal intensity confined to signal perimeter of normal amount of fluid on corresponding fluid-sensitive image.	Mild High signal intensity focally exceeding signal perimeter of physiological amount of joint fluid on corresponding fluid-sensitive image.	Moderate/severe High signal intensity diffusely involving one or both joint compartments.
B) Damage domain (possible total scores 0–5)				
Condylar flattening	Loss of the round or slightly angular shape of the condylar head, viewed in the sagittal-oblique plane.	Absent Round/slightly angular shape of condyle.	Mild Extent of flattening involves part of the surface of the condyle.	Moderate/severe Extent of flattening involves the entire surface of the condyle, or loss of height of the condyle.
Erosions	Any irregularity or break of the bony joint surfaces leading to the loss of the smooth continuous outline of the bone.	Absent No irregularities or deep breaks.	Mild Presence of irregularities involving only part of the articular surface of the condyle.	Moderate/severe Presence of deep breaks in the subchondral bone seen in two planes, or irregularities involving the entire articular surface of the condyle.
Disk abnormalities	Any abnormality of the articular disk, including flattening, displacement or destruction.	Absent	Present	

Current version, adapted and updated from references [15, 33]

**Table 2:** Progressive scoring system for assessing inflammation and osseous deformity of temporomandibular joint by magnetic resonance imaging

Inflammation		Osseous deformity	
Grade 0	No inflammation: No or small amounts of joint fluid in any recess, with ≤ 1 mm width. No enhancement or enhancement confined to physiological joint fluid.	Grade 0	Normal shape of temporal bone and mandibular condyle according to age: S-shaped articular eminence/glenoid fossa. Round condyle (young patient). Less rounded, more angular appearing condyle (older patient). Smooth subchondral bone contour.
Grade 1	Mild inflammation: Extension of joint enhancement exceeds that of physiological joint fluid but does not involve entire joint compartment and/or presence of bone marrow oedema.	Grade 1	Mild flattening of the mandibular condyle and/or temporal bone.
Grade 2	Moderate inflammation: Joint enhancement involves entire joint compartment or there is an enhancing joint effusion.	Grade 2	Moderate flattening of the mandibular condyle and/or temporal bone.
Grade 3	Severe inflammation: Detectable synovial thickening in addition to increased joint enhancement or effusion.	Grade 3	Severe flattening of the mandibular condyle with loss of height, and/or completely flat temporal bone, and/or presence of small erosions/irregularities.
Grade 4	Joint space filled with and enlarged by pannus.	Grade 4	“Destruction” of temporomandibular joint by large erosions, fragmentation of the mandibular condyle, intra-articular ossification or bone apposition on mandibular condyle or temporal bone.

Adapted and modified from references [2, 11]

### Bone marrow oedema

**Fig. 6:**
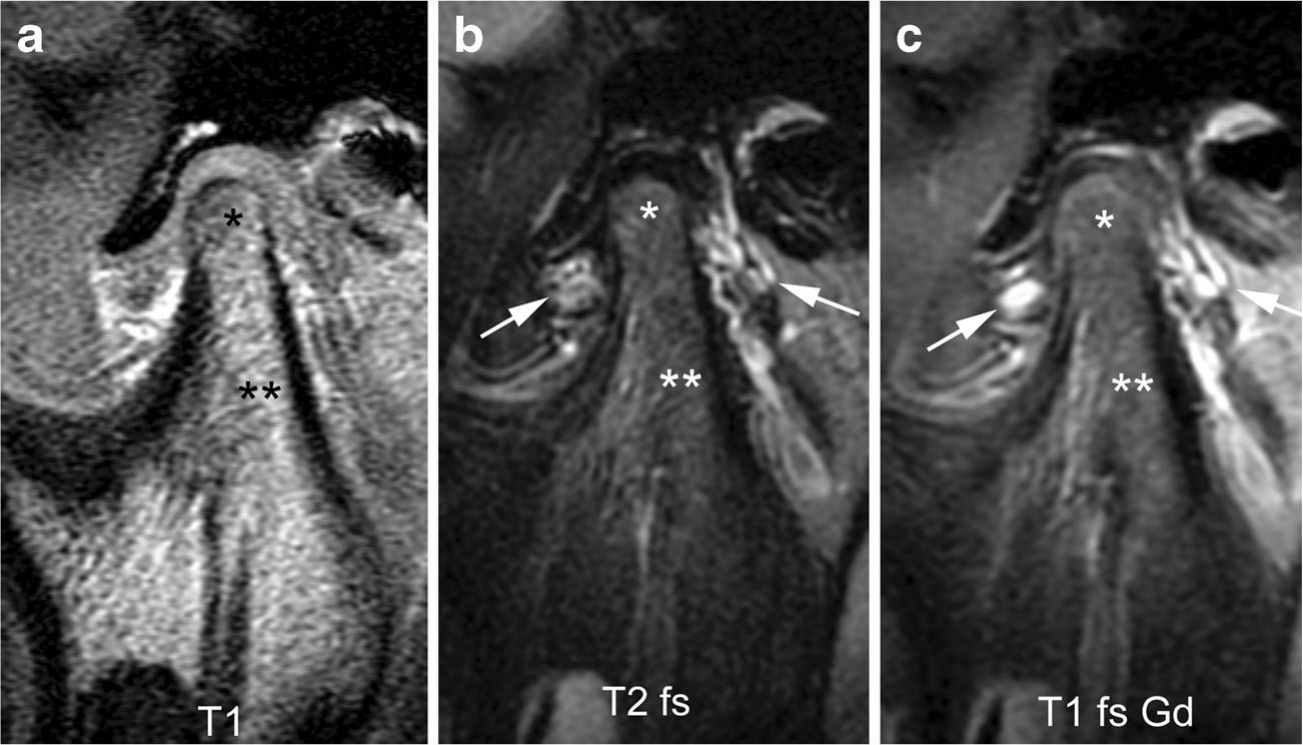
Normal bone marrow signal and enhancement (grade 0) in a 14-year-old boy. **a**-**c** Sagittal oblique T1-weighted (TR/TE 300/4.2 ms, flip angle 80°) gradient echo image without fat saturation (**a**), T2-weighted (TR/TE 5,400/77 ms) fat saturated image (**b**) and postcontrast fat-saturated T1-weighted (TR/TE 670/10 ms) fast spin echo images (**c**) show the marrow space of the mandibular condyle (*) with isointense signal compared to that of the mandibular ramus (**). Note prominent veins surrounding the joint (arrows in **b**,**c**) and slightly increased enhancement of the posterior upper joint compartment. For this age, the condyle appears rather round and lacks an anterior tilt of the mandibular neck. T1 T1-weighted, T1 fs Gd contrast-enhanced fat saturated T1-weighted, T2 fs fat saturated T2-weighted

**Fig. 7:**
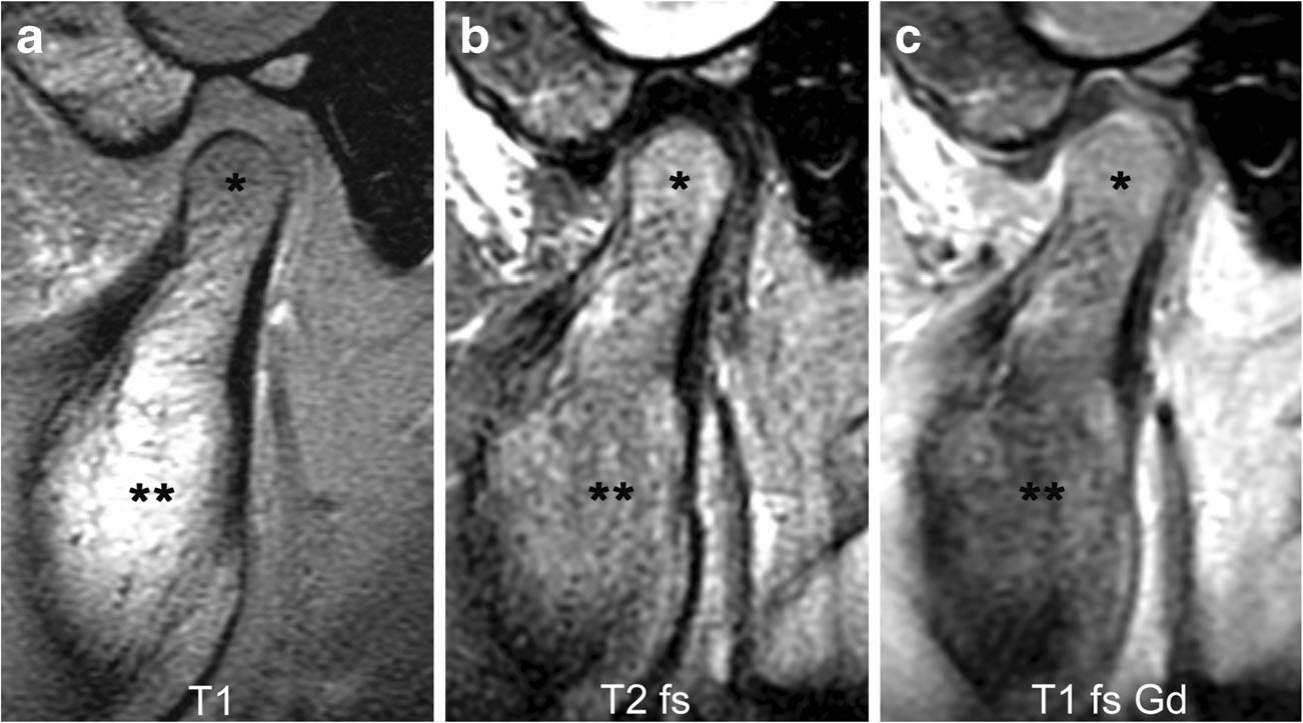
Bone marrow oedema and enhancement (grade 1) of the mandibular condyle in a 9-year-old boy. **a**-**c** When compared to the mandibular ramus (**) there is lower signal from the mandibular condyle (*) on the T1-weighted gradient echo image without fat saturation (TR/TE 300/4.2 ms, flip angle 80°, **a**), and slightly increased signal on fat-saturated T2-weighted image (TR/TE 5,400/77 ms, **b**) and on postcontrast fat-saturated T1-weighted image (TR/TE 670/10 ms, **c**) images, Note mild joint enhancement with high signal intensity (hyperintense to muscle) in the posterior superior joint recess and lower joint compartment on postcontrast fat-saturated T1-weighted image (**c**)

Because MRI signal characteristics of bone marrow vary with the proportions of haematopoietic and fatty marrow, presence of oedema in the condyle is assessed by comparing its marrow space signal intensity to that of the mandibular ramus. Bone marrow oedema of the condyle is defined as hypointense signal on T1-weighted images without fat saturation and hyperintense signal on fluid-sensitive images in comparison to that of the ramus. It can be graded as absent (grade 0) (Figs. 2, 6 and 11) or present (grade 1) (Figs. 7, 9, 12, 15 and 22).

### Bone marrow enhancement

The degree of normal marrow enhancement also depends on the proportions of haematopoietic and fatty marrow, with red marrow showing more enhancement than yellow marrow. Increased enhancement of the marrow in the condyle is defined as hyperintense signal compared to that of the mandibular ramus on fat-saturated T1-weighted images. Increased bone marrow enhancement can be graded as absent (grade 0) (Figs. 3, 6, and 11) or present (grade 1) (Figs. 7, 12, 13 and 22).

### Joint effusion

**Fig. 8:**
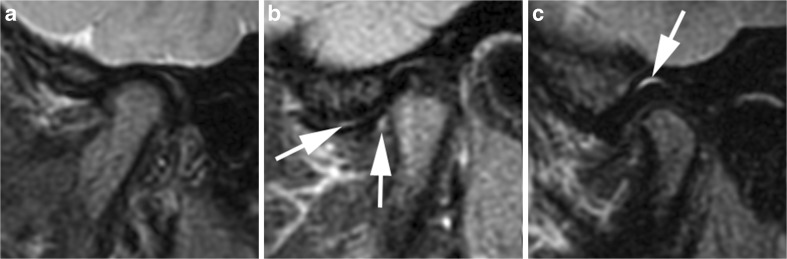
No joint effusion (grade 0). **a**-**c** Sagittal oblique fat-saturated T2-weighted fast spin echo images (TR/TE 5,400/77 ms). There is no high signal intensity fluid within the joint compartments in a 3-year-old boy (**a**). There is a normal amount of fluid in the anterior recesses (arrows in **b**) in a 14-year-old boy and in the superior joint compartment (arrow in **c**) in a 14-year-old girl

**Fig. 9:**
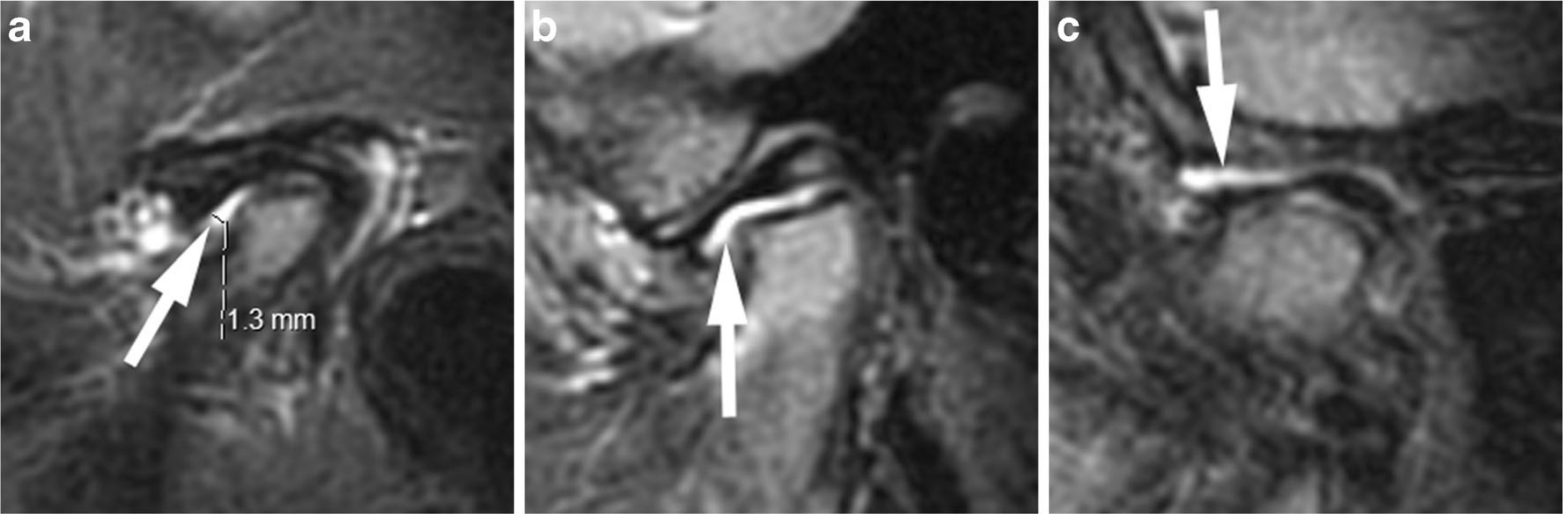
Small joint effusion (grade 1). **a**-**c** Sagittal oblique fat-saturated T2-weighted fast spin echo images (TR/TE 5,400/77 ms) show high signal intensity (arrows) of >1-mm and ≤2-mm thickness within the joint spaces in the lower anterior recess in a 6-year-old girl (**a**), in the lower joint compartment (**b**) and upper joint compartment (**c**) in a 5-year-old boy. Note mild flattening of condyle in (**a**), and moderate/severe flattening of the condyle in (**b**) and (**c**)

**Fig. 10:**
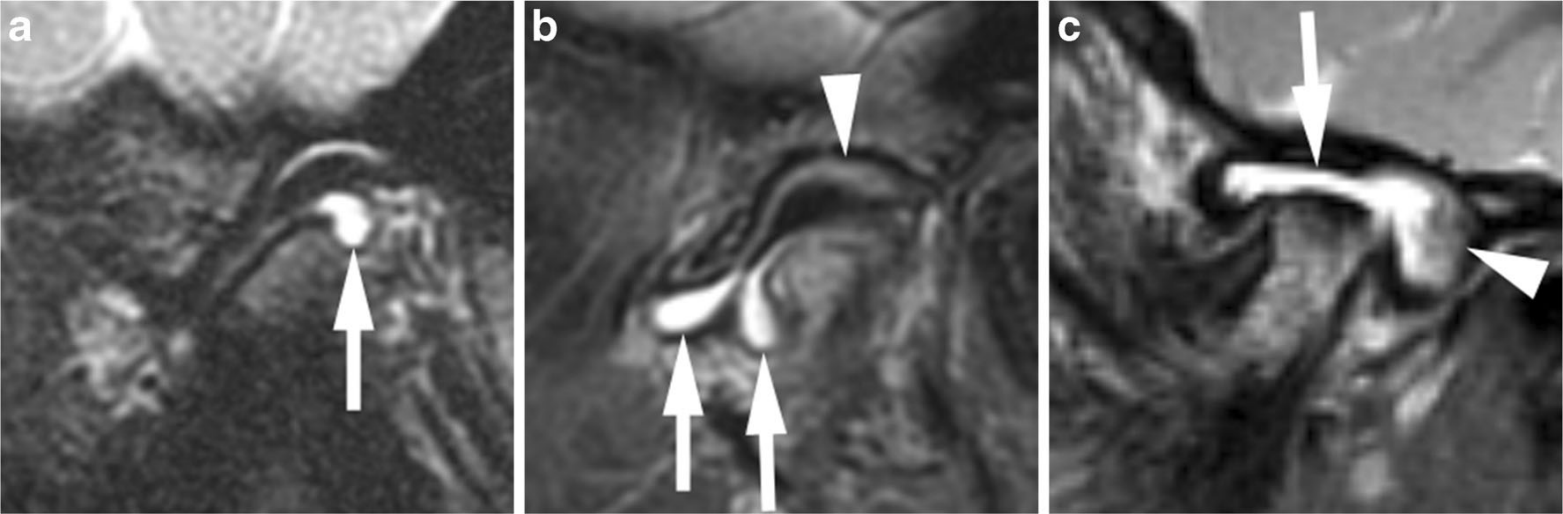
Large joint effusion (grade 2) on sagittal oblique images. **a**-**c** Effusion exceeding 2-mm width (arrows) on fat-saturated T2-weighted fast spin echo images (TR/TE 5,400/77 ms) in the posterior recess in a 15-year-old girl (**a**), in the anterior recesses in a 13-year-old boy (**b**) and with bulging of the upper joint compartment in a 7-year-old girl (**c**). Note thickened synovium with intermediate signal intensity (arrowheads) on these posteriorly in (**b**) and (**c**)

**Fig. 11:**
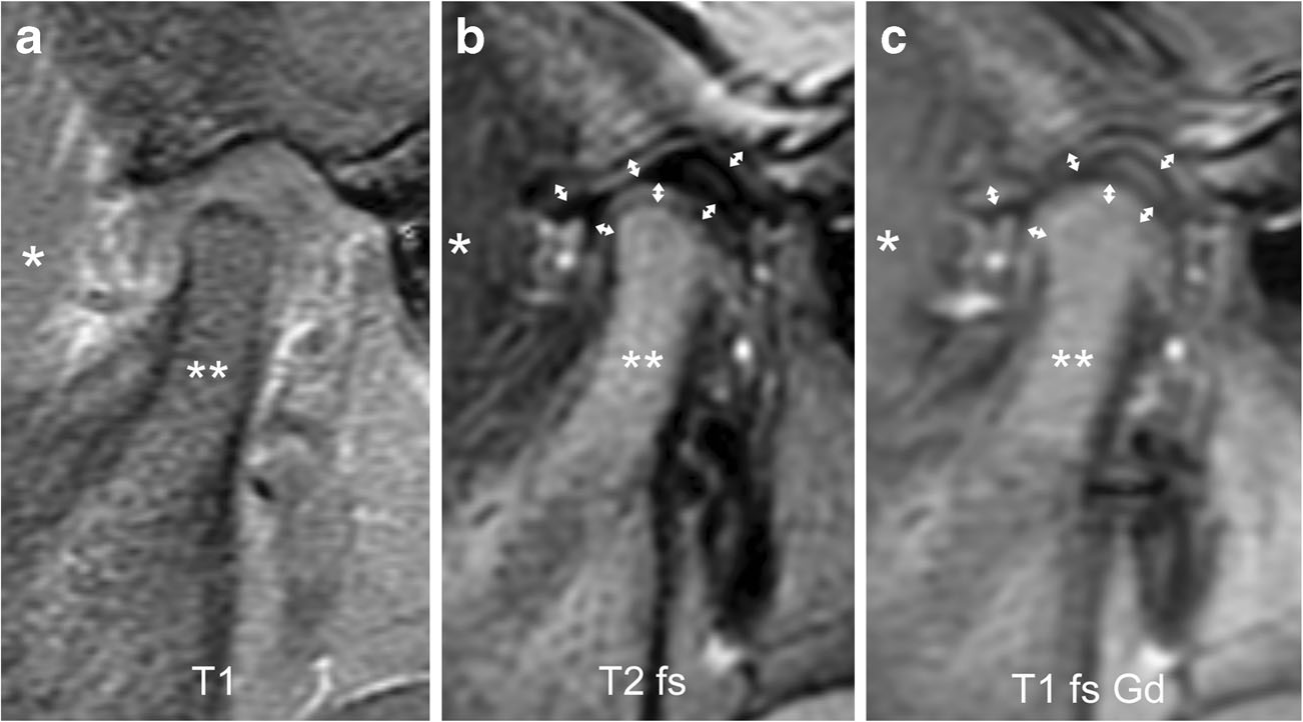
No synovial thickening (grade 0) and no joint enhancement (grade 0) on sagittal oblique images in a 3-year-old boy. **a** There is a large proportion of haematopoietic marrow in the mandible, which is shown as low signal intensity (**) similar to that of muscle tissue (*) in an unenhanced T1-weighted gradient echo image without fat saturation (T1, TR/TE 300/4.2 ms, flip angle 80°). **b**-**c** The width of both joint compartments (including articular cartilage, synovium and joint space) between the bony articular surface and disk is smaller than 1 mm (small double arrows) on fat-saturated T2-weighted image (TR/TE 5,400/77 ms, **b**) and on contrast-enhanced fat-saturated T1-weighted image (TR/TE 670/10 ms, **c**). Postcontrast signal intensity of the joint spaces is similar to that muscle (*). There is higher signal intensity compared to that of muscle (*) in the fluid-sensitive image (**b**) and postcontrast fat-saturated T1-weighted image (c). T1 T1-weighted, T1 fs Gd contrast-enhanced fat saturated T1-weighted, T2 fs fat saturated T2-weighted

**Fig. 12:**
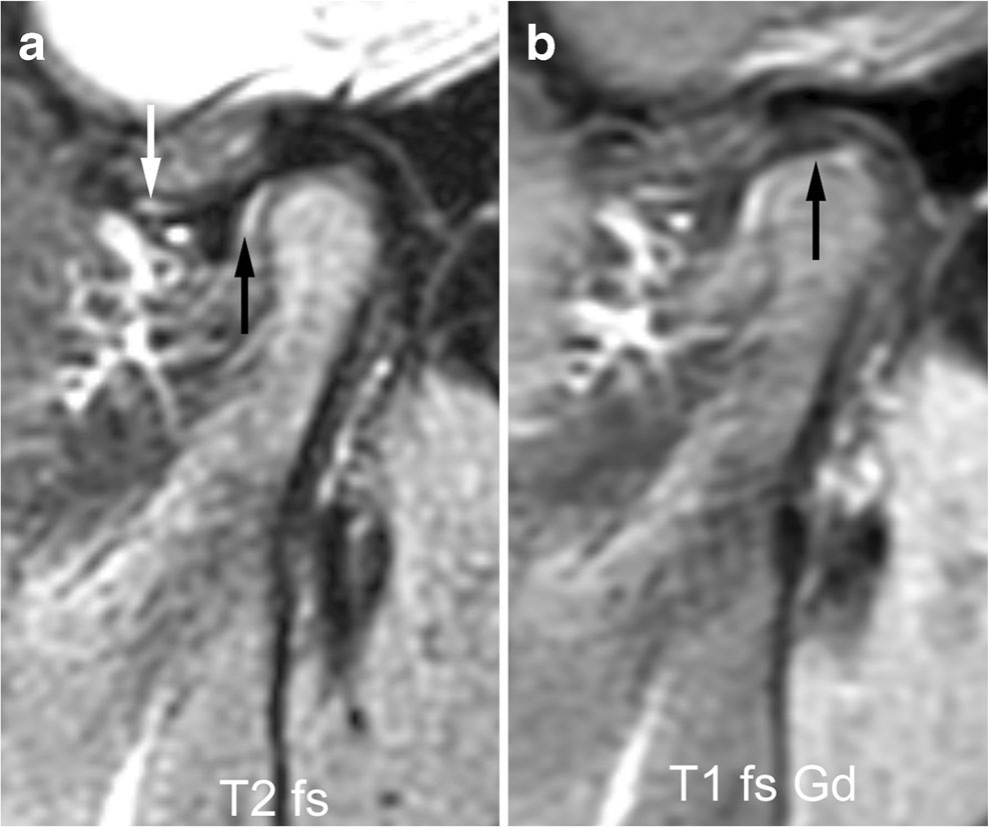
No synovial thickening (grade 0) but mild increase of joint enhancement (grade 1) on sagittal oblique images in a 6-year-old boy. **a** Fat saturated T2-weighted image (TR/TE 5,400/77 ms) shows normal amount of joint fluid in upper and lower anterior recesses (arrows). **b** Postcontrast T1-weighted fat-saturated image (TR/TE 670/10 ms) shows high signal intensity, isointense to that of veins, in both joint compartments. At the midportion of the lower joint compartment (arrow) this exceeds the extent of high signal intensity seen in (**a**). Note mild bone marrow oedema and enhancement of the normally shaped mandibular condyle. T1 fs Gd contrast-enhanced fat saturated T1-weighted, T2 fs fat saturated T2-weighted

**Fig. 13:**
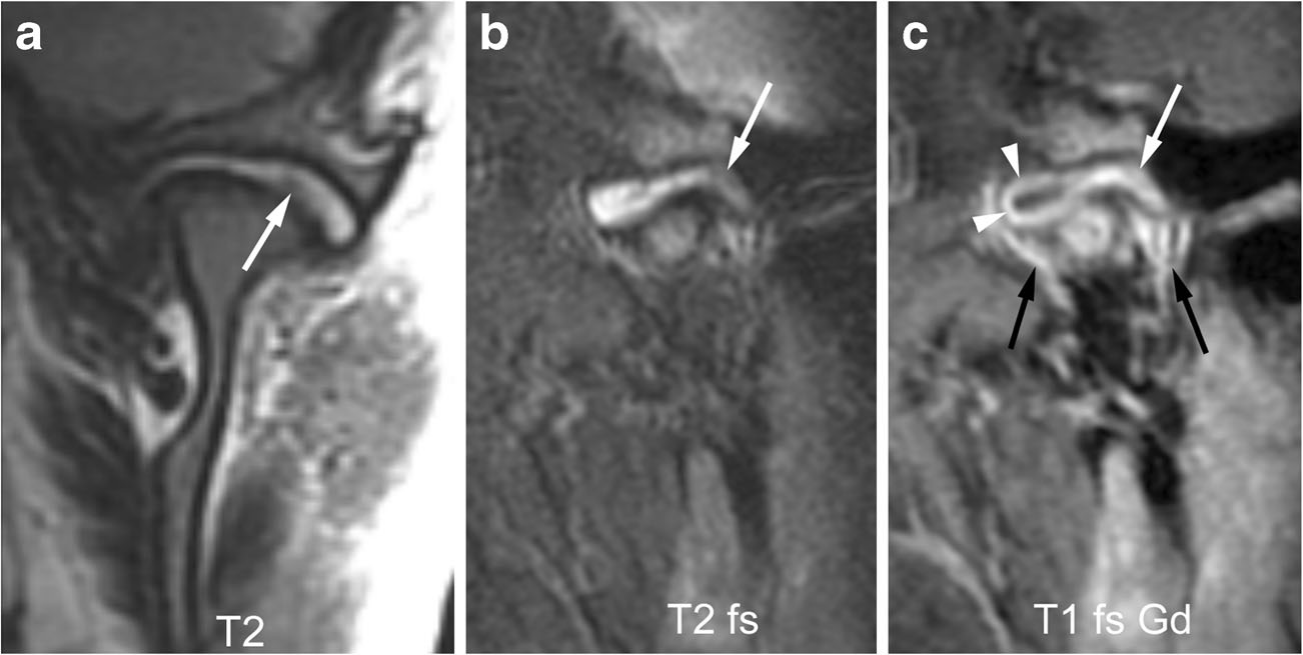
Mild synovial thickening (grade 1) and severe joint enhancement (grade 2). **a**-**b** Coronal T2-weighted image (TR/TE 2,600/110 ms, a) and sagittal oblique fat-saturated T2-weighted image (TR/TE 5,400/77 ms, **b**) show a large effusion with high signal intensity and some nodular areas of synovial thickening with intermediate signal intensity in posterior portion of the upper joint compartment (arrows). **c** Corresponding early postcontrast fat-saturated T1-weighted (TR/TE 670/10 ms) image demonstrates enhancement of the synovium (white arrow) and peripheral joint fluid (arrowheads) involving the entire upper compartment, with same signal intensity as surrounding veins (black arrows). T1 fs Gd contrast-enhanced fat saturated T1-weighted, T2 T2-weighted, T2 fs fat saturated T2-weighted

**Fig. 14:**
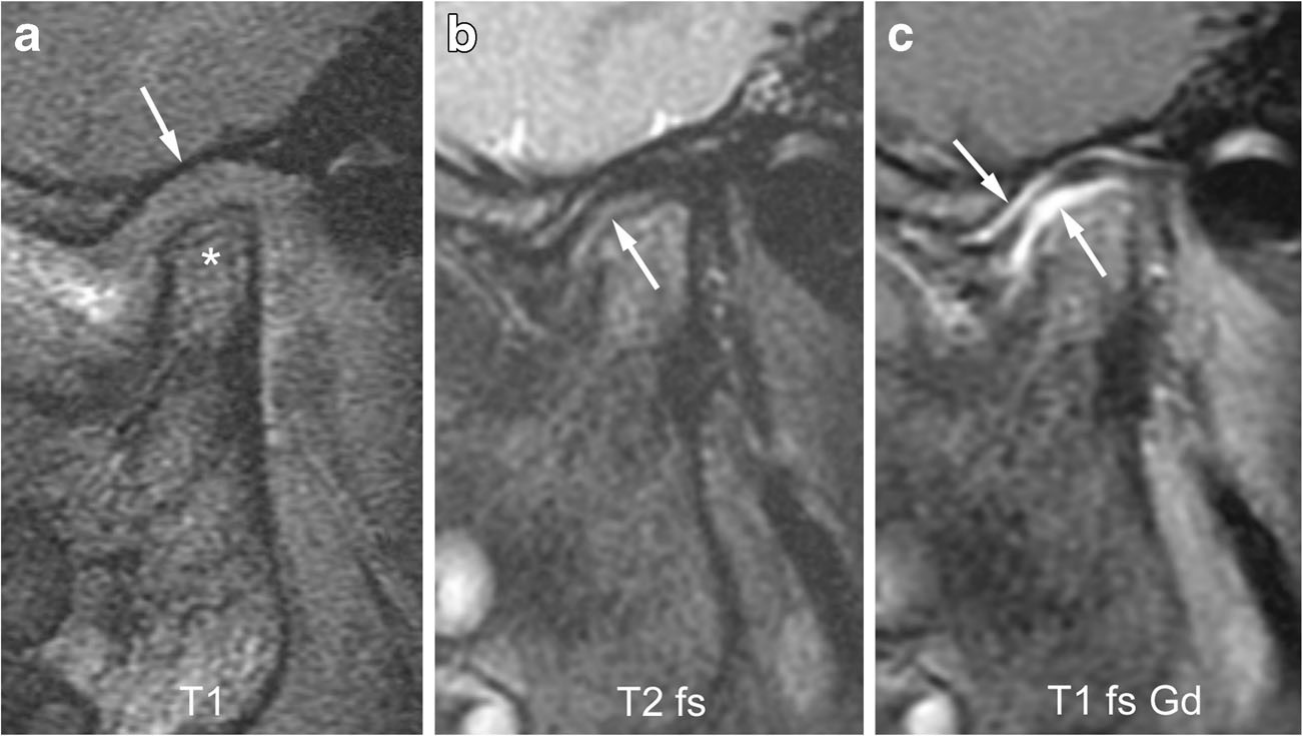
Mild synovial thickening (grade 1) and severe joint enhancement (grade 2) on sagittal oblique images in a 5-year-old boy. **a** There moderate flattening of condyle (*) and mild flattening of temporal bone (arrow) on T1-weighted image (TR/TE 300/4.2 ms, flip angle 80°, without fat saturation). **b** Fluid-sensitive, fat-saturated T2-weighted fast spin echo image (TR/TE 5,400/77 ms) shows tissue with intermediate signal intensity exceeding 1-mm width in lower joint space (arrow). **c** Postcontrast fat-saturated T1-weighted image (TR/TE 670/10 ms) shows high signal intensity (arrows) involving both the upper and lower joint compartments. T1-weighted, T2 T2-weighted, T2 fs fat saturated T2-weighted

Increased amounts of synovial fluid with high signal intensity similar to that of cerebrospinal fluid on fluid-sensitive sequences are considered a joint effusion. Joint effusion is graded as absent (grade 0, normal amount of intraarticular fluid ≤1 mm width in recess) (Figs. 2–4, 6–8, 11, 12, 14, 16 and 22), small (grade 1, fluid >1 mm and ≤2 mm width in recess or fluid involving entire one or both joint compartments) (Figs. 9 and 24), or large (grade 2, fluid >2 mm width with bulging of joint compartments) (Figs. 10 and 13). The maximal short-axis thickness of the largest effusion pocket at any joint recess is measured on sagittal oblique fluid-sensitive images, which determines the thresholds for each grade.

### Synovial thickening

**Fig. 15:**
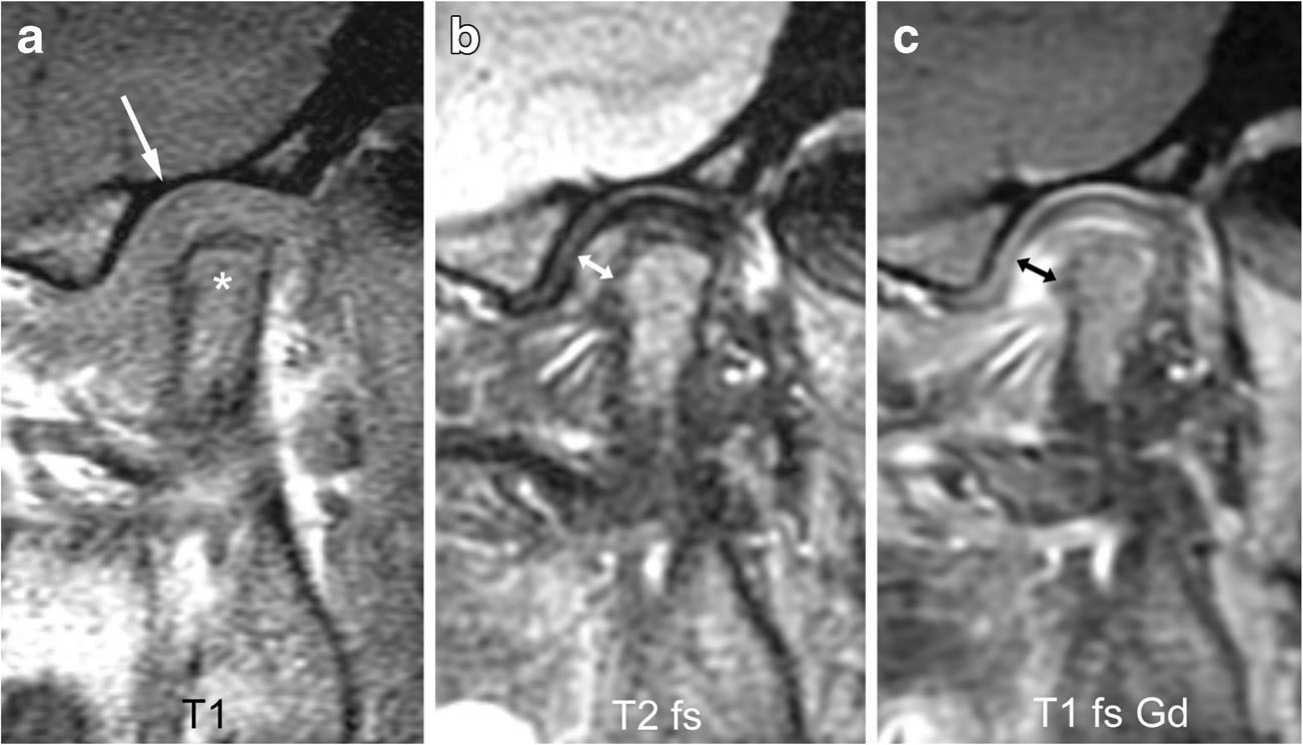
Severe synovial thickening (grade 2) and severe joint enhancement (grade 2) on sagittal oblique images in a 11-year-old girl. **a** There is moderate flattening of the condyle (*) and mild flattening of the temporal bone (arrow) on T1-weighted image (TR/TE 300/4.2 ms, flip angle 80°). **b** Thickened synovium (arrow) is shown in the anterior inferior portion of the joint space as intermediate signal intensity tissue exceeding 2-mm width on fat saturated T2-weighted fast spin echo (TR/TE 5,400/77 ms). **c** There is strong enhancement of the thickened synovium (arrow) but also the entire lower joint space and the posterior half of the upper joint space are shown as high signal intensity on postcontrast fat-saturated T1-weighted image (TR/TE 670/10 ms). T1 T1-weighted, T1 fs Gd contrast-enhanced fat saturated T1-weighted, T2 fs fat saturated T2-weighted

**Fig. 16:**
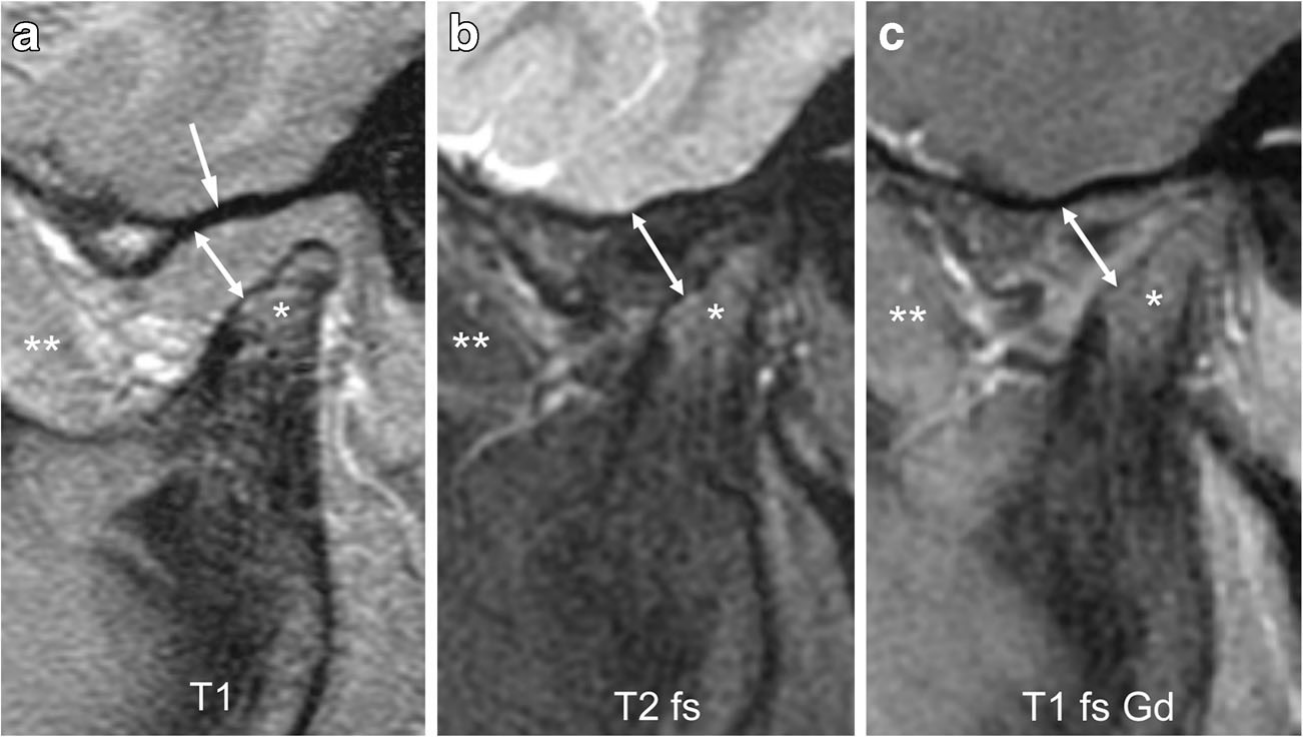
Severe synovial thickening (grade 2) but no increased joint enhancement (grade 0) on sagittal oblique images in an 8-year-old girl. **a**-**c** There is moderate flattening of the mandibular condyle (*) and mandibular fossa (arrow in **a**), and joint space expansion (double arrow) on T1-weighted image (TR/TE 300/4.2 ms, flip angle 80°, **a**), fat-saturated T2-weighted fast spin echo image (TR/TE 5,400/77 ms, **b**) and on postcontrast fat-saturated T1-weighted image (TR/TE 670/10 ms, **c**). The synovium is isointense to muscle (**) on all images. T1 T1-weighted, T1 fs Gd contrast-enhanced fat saturated T1-weighted, T2 fs fat saturated T2-weighted

Both joint compartments may be identified on fast spin echo images as thin lines with intermediate signal intensity between the subchondral bone surface and articular disk (Fig. 11). In the central portions of the temporomandibular joint, these lines comprise articular fibrocartilage, cartilaginous layers of the growth zone and joint space, but no synovium. The normal synovium, which is located in the joint recesses, is not perceptible on MRI. Thickened synovium is evident on fluid-sensitive images as tissue with intermediate signal intensity (hyperintense to muscle but hypointense to fluid) that widens the respective joint compartment. Synovial thickening is graded as absent (grade 0, no perceptible synovium with joint compartment width ≤1 mm) (Figs. 11 and 12), mild (grade 1, >1 mm and ≤ 2 mm width) (Figs. 10, 13, 14 and 22), or severe (grade 2, >2 mm width) (Figs. 10, 15 and 16). Synovial thickness should be measured in the maximal short-axis dimension on sagittal oblique fluid-sensitive images perpendicular to the orientation of the joint space and condylar head surface.

### Joint enhancement

In an inflamed temporomandibular joint, hyperaemia and augmented diffusion of contrast agents into the synovial fluid lead to increased enhancement of the synovium and joint space [26, 30, 34]. Thickened synovium and joint fluid are considered to enhance if their signal intensity exceeds that of muscle or is similar to that of veins on postcontrast fat-saturated T1-weighted images. Because the gadolinium-based contrast agent quickly diffuses into the synovial fluid, which results in a gradual signal intensity increase eventually involving the entire joint space, judgment of joint enhancement always needs to be performed at the same time following contrast administration. Hence, due to the fact that both synovium and joint fluid present with high signal after contrast administration, we collectively use the term “joint enhancement” to represent enhancement of adjacent synovial lining and joint fluid. For grading the extent of joint enhancement, it is recommended to assess the signal intensity of the joint space and synovium on sagittal oblique fat-saturated T1-weighted fast spin echo images acquired within 4 min after contrast injection. Joint enhancement is considered normal (grade 0) when there is no high signal intensity in joint compartments (Figs. 11 and 16) or there is only high signal intensity confined to small normal amounts of joint fluid as determined on corresponding unenhanced fluid-sensitive images (Figs. 3 and 4). When the signal intensity of a joint compartment or thickened synovium is similar to adjacent muscle in the early postcontrast phase, it is considered to not show increased enhancement (Figs. 11 and 16). Mild joint enhancement (grade 1) is present when the high signal intensity exceeds that of small normal amounts of joint fluid (Figs. 6, 7 and 12). Severe joint enhancement is present when the high signal intensity diffusely involves one or both joint compartments (Figs. 5, 13, 15 and 22).

### Osseous deformity - temporal bone and condylar flattening

**Fig. 17:**
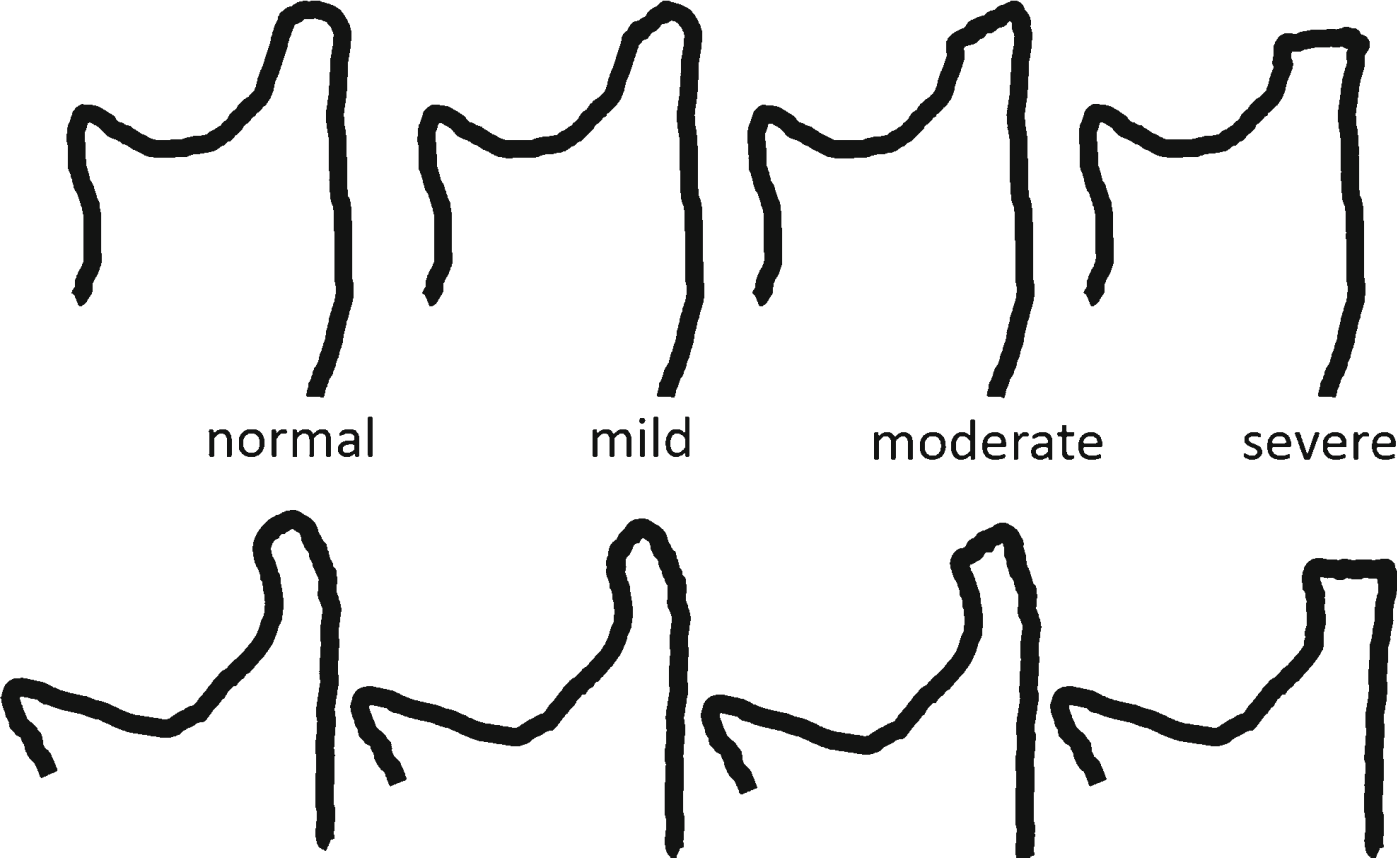
Grades of condylar flattening. Schematic drawing showing different degrees of condylar flattening viewed in the sagittal oblique plane, in a young child (upper row) up to about 5 years of age and in an older child (lower row)

**Fig. 18:**
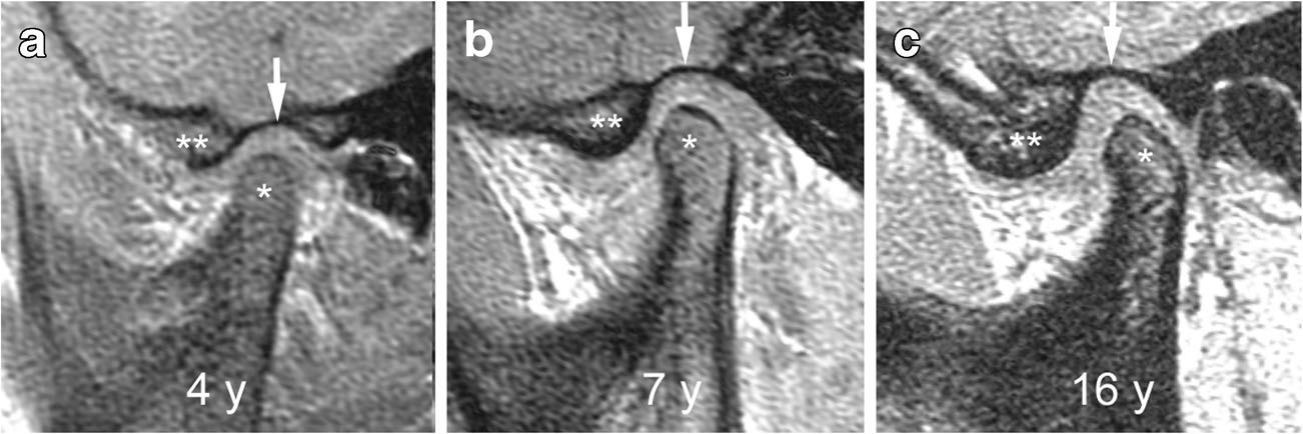
Normal shape of mandibular condyle (grade 0) and temporal bone on sagittal oblique T1-weighted gradient echo images (TR/TE 300/4.2 ms, flip angle 80°, without fat saturation). **a**-**c** Images from three different children show the changing configuration of the mandibular condyle (*), the articular eminence (**) and the glenoid fossa (arrows) among a 4-year-old (**a**), a 7-year-old (**b**) and a 16-year-old (**c**). In a 4-year-old child (**a**) the superior articular contour is round with a straight condylar neck. With increasing age and growth, the condylar neck gains an anterior tilt and the head appears more angular, with less rounding of the anterior-superior joint surface. The articular eminence gets larger resulting in a deeper glenoid fossa

**Fig. 19:**
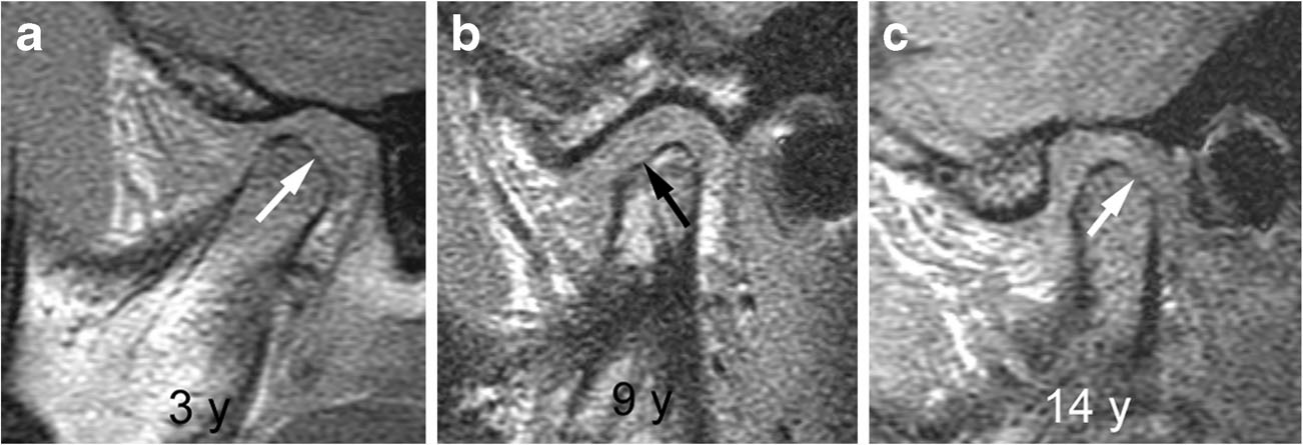
Mild condylar flattening (grade 1). **a**-**c** Sagittal oblique T1-weighted gradient echo images (TR/TE 300/4.2 ms, flip angle 80°, without fat saturation) from different children obtained at 3 years (**a**), 9 years (**b**) and 14 years (**c**) show flattening involving part of the condylar surface (arrows)

**Fig. 20:**
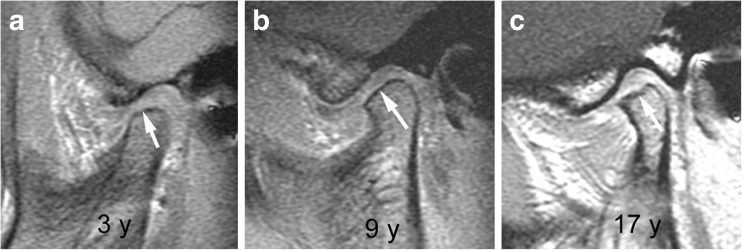
Moderate condylar flattening (grade 2). **a**-**c**. Sagittal oblique T1-weighted gradient echo images (TR/TE 300/4.2 ms, flip angle 80°, without fat saturation) from different children obtained at 3 years (**a**), 9 years (**b**) and 17 years (**c**) show flattening involving the entire surface of the condyle with preserved anterior angulation (arrows)

**Fig. 21:**
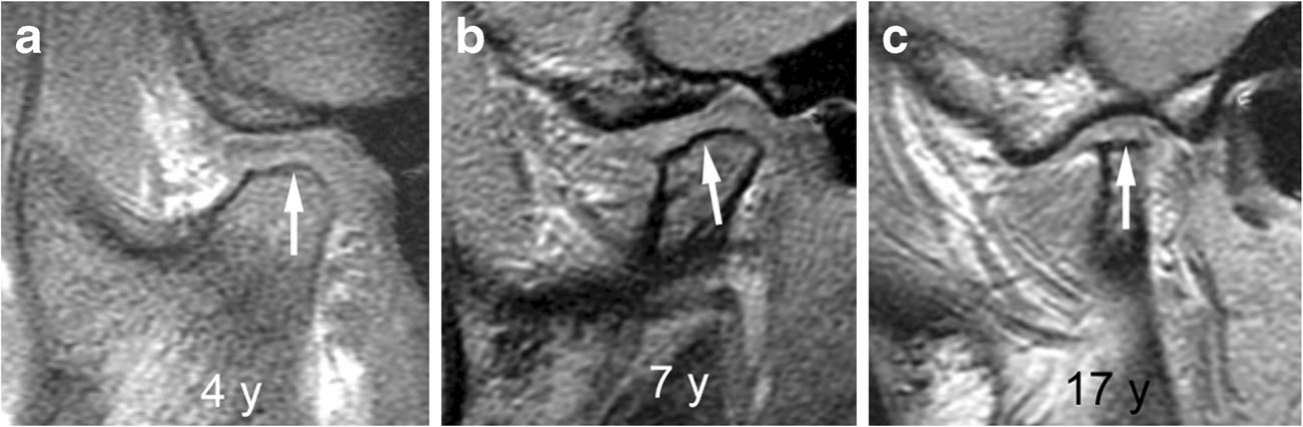
Severe condylar flattening (grade 2). **a**-**c** Sagittal oblique T1-weighted gradient echo images (TR/TE 300/4.2 ms, flip angle 80°, without fat saturation) from different children obtained at 4 years (**a**), 7 years (**b**) and 17 years (**c**) show flattening involving the entire surface of the condyle and horizontally oriented joint surface (arrows)

**Fig. 22:**
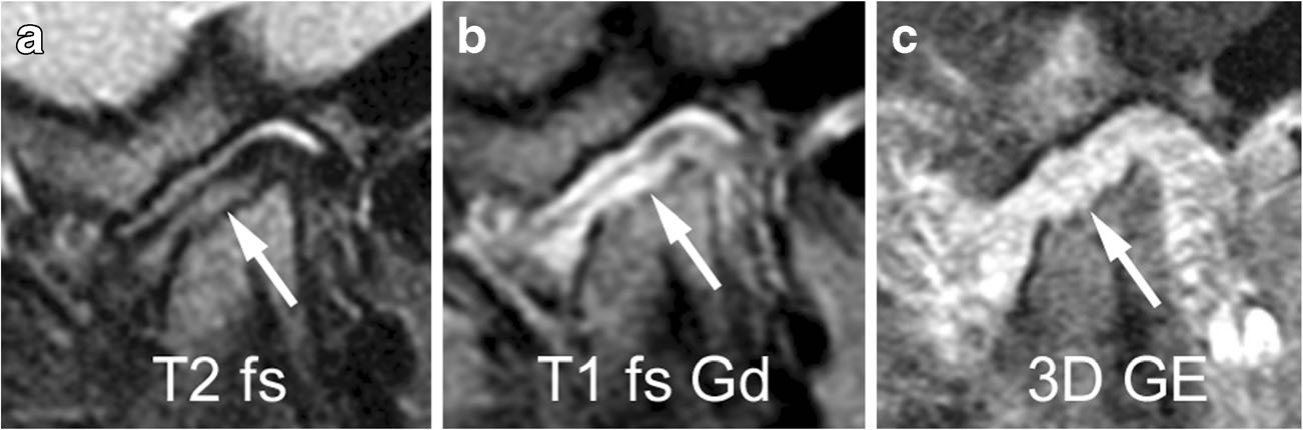
Small erosions in a 7-year-old girl on sagittal oblique images. **a**-**c** Irregularity of the bony articular surface (arrows) with small breaks of the subchondral bone is evident on fat saturated fast spin echo image (TR/TE 5,400/77 ms, **a**) and on fat-saturated postcontrast T1-weighted image (TR/TE 670/10 ms), but is best delineated on gradient echo image (TR/TE 10/4.2 ms, flip angle 20°, **c**). For assessing the extent of these erosions (part of condylar surface versus entire articular surface), all successive images covering the condyle from lateral to medial need to be viewed. Note bone marrow oedema, mild synovial thickening and severe joint enhancement. 3D GE volumetric T1-weighted gradient echo, T1 fs Gd contrast-enhanced fat saturated T1-weighted, T2 fs fat saturated T2-weighted

A flattened appearance of both the temporal bone and mandibular condyle surfaces is characteristic of temporomandibular joint involvement in juvenile idiopathic arthritis [1]. While loss of the normal s-shape of the temporal bone is likely due to arrested development of the articular eminence, flattening of the mandibular condyle may be the result of growth disturbance, destruction and remodeling due to inflammation [1]. The mandibular condyle typically shows diminished craniocaudal and lateromedial dimensions, while the anteroposterior dimension may be increased, resulting in a flat appearance on sagittal oblique views. With some experience, deformity of the temporal bone and mandibular condyle can be qualitatively graded as mild, moderate or severe [2]. Similar to radiography [35], the degree of straightening, or loss of the normal round or slightly angular shape of the condylar head, can be judged by considering the angle of flattening and loss of condylar height on sagittal oblique images (Figs. 17, 19–21). In an older child, resemblance of the condyle to that of an infant should also be considered a deformity [1] (Fig. 6). For the current version of the additive scoring system, the extent of condylar flattening is graded as absent (grade 0) when there is a normal round or slightly angular contour (Figs. 7, 12 and 18), as mild (grade 1) when only parts of the condyle are involved (Figs. 3, 11 and 19) or as moderate/severe when the whole condyle is involved (Figs. 12, 14, 15, 20–22).

**Fig. 23:**
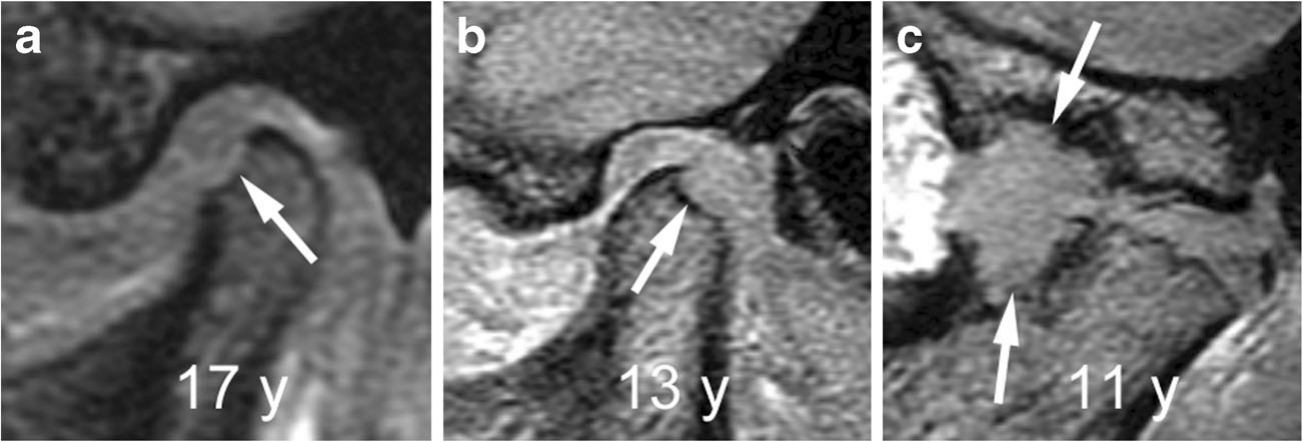
Large erosions. **a**-**c** Sagittal oblique gradient echo images (TR/TE 10/4.2 ms, flip angle 20°) from different children show large defect (arrow) of the anterior condylar surface in a 17-year-old (**a**), of the posterior condylar surface (arrow) in a 13-year-old (**b**) and involving both a severely deformed mandibular condyle and the articular eminence of the adjacent temporal bone (arrows) in an 11-year-old (**c**)

**Fig. 24:**
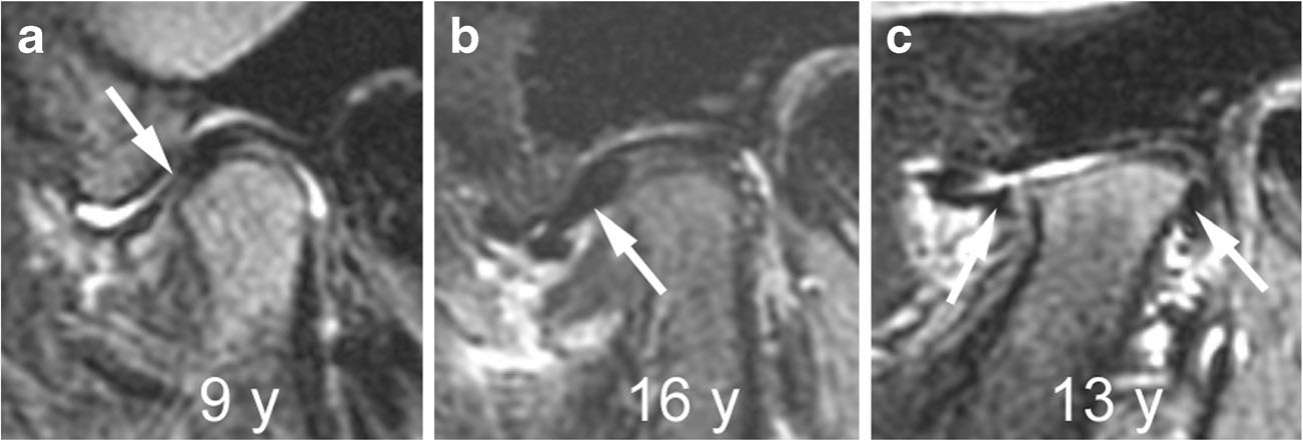
Abnormalities of articular disk. **a**-**c** Sagittal oblique fat-saturated T2-weighted fast spin echo images (TR/TE 5,400/77 ms) from different children show a flat disk (arrow) in a 9-year-old (**a**), an anteriorly dislocated disk (arrow) in a 16-year-old (**b**) and a perforated disk with peripheral remnants (arrows) seen in the anterior and posterior joint recesses in a 13-year-old (**c**)

### Erosion

As there is no general definition of erosions on MRI, we define erosions in the temporomandibular joint as irregularities, depressions or breaks of the subchondral low intensity lines that demarcate the articular surfaces. While deep breaks probably represent true erosions containing inflammatory pannus, small irregularities of the articular surface more likely represent defects or disturbed mineralisation of subchondral bone with preserved overlying fibrocartilage. The degree of erosions is graded as absent (grade 0) when the outline of subchondral bone is smooth and continuous, as mild (grade 1) when small irregularities involve parts of the condylar surface (Fig. 22), and as severe (grade 2) when small irregularities involve the entire condylar surface, or when deep breaks are seen in two planes (Fig. 23).

### Disk abnormality

Abnormalities of the articular disk are well recognised in patients with juvenile idiopathic arthritis and can coexist with any degree of synovial or osseous abnormality [6, 36, 37]. The most common findings are flat or thin disk (i.e. loss of normal biconcave shape), perforation or fragmentation, and displacement. Disk abnormality is graded as absent or present (Figs. 3 and 24).

## Essential MRI protocol

The minimal requirement for grading temporomandibular joint involvement as described in this article is a set of four sequences covering both joints. It includes unenhanced T1-weighted and fluid-sensitive images for assessing bone marrow, joint fluid and synovial thickening as well as fat-saturated T1-weighted images in two planes obtained after intravenous injection of gadolinium-based contrast agent for assessing bone marrow and joint enhancement. The precontrast T1-weighted images should be acquired without fat suppression either by fast spin echo or fast spoiled gradient echo sequences. The fast spin echo technique is more reliable for assessing bone marrow signal intensity, while the gradient echo technique allows for superior delineation of the subchondral bone surface and shape of the temporomandibular joint [2, 18]. Fluid-sensitive images can be obtained either by T2-weighted fast spin echo techniques employing fat saturation or fast short tau inversion recovery STIR techniques. While fast spin echo images allow for more contrast resolution and signal-to-noise ratio at a given imaging time, homogenous saturation of fat signal may not always be achieved with spectral fat saturation pulses. Alternatively, a Dixon technique allows very homogenous suppression of signal from fat and provides higher contrast-to-noise ratio than a fast STIR sequence. Selection of the optimal fluid-sensitive sequence will depend on the availability and performance on a given MRI system.

Measuring and quantifying contrast enhancement by MRI necessitates either a dynamic technique or two T1-weighted sequences obtained before and after contrast administration [23, 26, 27, 30, 38]. Image sets with identical parameters can be subtracted and hence would be highly sensitive for enhancement. However, presence of enhancement can be judged by comparing the signal intensity of a structure on fat-saturated T1-weighted images to that of other vascularised tissues known to take up gadolinium-based contrast agents (i.e. muscle tissue, bone marrow or veins) [13, 23, 34, 39]. The extent and intensity of enhancement should be graded on the first images obtained immediately after injecting contrast agent [2] because there is almost immediate and ongoing diffusion of the contrast agent into the small temporomandibular joint leading to gradually increasing signal intensity of the entire joint space over time. Sagittal oblique sectioning of the temporomandibular joint, aligned perpendicular to the long axis of the mandibular condyle and parallel to the mandibular ramus, is generally accepted as the most valuable imaging plane for displaying the synovial compartments with the anterior and posterior recesses, the articular disk and the retrodiskal tissue on a single image. For the additive scoring system, sagittal oblique fluid-sensitive images are required for measuring the width of joint effusion and synovial thickness as thresholds for grading. In addition, obtaining fluid-sensitive images and the first set of contrast-enhanced images in the same plane and at identical positions allows for investigation of the extent of contrast-enhancement within the synovial joint compartments. Hence, contrast-enhanced images should be first obtained in the sagittal oblique plane, followed by images in the coronal or coronal oblique planes. A comprehensive morphological and functional evaluation of the articular disk is best performed with proton density weighted fast spin echo sequences with closed and open mouth sagittal oblique views. However, shape, integrity and position of the articular disk are also apparent on the suggested fluid-sensitive and postcontrast fat-saturated T1-weighted fast spin echo images. Height of the mandibular ramus can be accurately measured on maximum intensity projections constructed from sagittal oblique 3-D gradient echo acquisitions covering each mandibular ramus [11, 40]. From all proposed MRI sequences, these images are also the best for assessing the osseous configuration (shape of condyle and temporal bone) and extent of bony erosions [18].

For delineation of small amounts of synovial fluid, thickened synovium, articular disk and bony erosions in the small temporomandibular joint, spatial resolution needs to be higher than usually provided by brain MRI protocols. Optimally, the voxel size should be at or below 2x0.5x0.5 mm^3^ [6, 41, 42]. The impairments of signal-to-noise and contrast-to-noise ratios resulting from such small voxel size needs to be compensated by using dedicated temporomandibular joint coils or multichannel surface coils, by increasing the number of signal averages, or by imaging at a higher field strength. Imaging parameters of two MRI protocols from the authors’ institutions, one for a 1.5-Tesla scanner employing dual ring surface coils and another for a 3-Tesla scanner employing a 32-channel head coil are given in Online Resource 2.

## Conclusion

We have presented an atlas for grading temporomandibular joint arthritis according to current scoring systems. Systematic assessment of the level of inflammation, degree of osteochondral deformation, and growth of the mandibular ramus by MRI may aid in monitoring the course of temporomandibular joint arthritis and evaluating treatment options.

## Electronic supplementary material

ESM 1 (PDF 4994 kb)

ESM 2 (PDF 96 kb)
