# Supplementary material for: Temporomandibular joint atlas for detection and grading of juvenile idiopathic arthritis involvement by magnetic resonance imaging
Source: Pediatr Radiol. 2017 Nov 13;48(3):411–26. doi: 10.1007/s00247-017-4000-0 (PMC5823950; doi:10.1007/s00247-017-4000-0)
Supplement: Supplementary file 1 — (PDF 4994 kb) [file 247_2017_4000_MOESM1_ESM.pdf]

Identification and Grading of Juvenile Idiopathic Arthritis Related Changes in the Temporomandibular Joints in Contrast Enhanced Magnetic Resonance Imaging:

An Imaging Atlas

Revised and shortened educational poster (EDU-107), presented at the 60th Annual Meeting and Categorical Course of the Society of Pediatric Radiology - Vancouver, BC, Canada on May 18th, 2017

(Junhasavasdikul T, Kellenberger CJ, Tolend M, Doria AS (2017) Identification and Grading of Juvenile Idiopathic Arthritis Related Changes in the Temporomandibular Joints in Contrast Enhanced Magnetic Resonance Imaging: An Imaging Atlas. Pediatr Radiol 47 (Suppl 1):S111-112. doi:10.1007/s00247-017-3809-x)

Online Resource 1 to pictorial essay

"Temporomandibular Joint Atlas for Detection and Grading of Juvenile Idiopathic Arthritis Involvement by Magnetic Resonance Imaging", Pediatric Radiology

Christian J. Kellenberger, Thitiporn Junhasavasdikul, Mirkamal Tolend, Andrea S. Doria

Corresponding Author:

Christian J. Kellenberger, Department of Diagnostic Imaging, University Children's Hospital Zürich,

christian.kellenberger@kispi.uzh.ch

Components of Temporomandibular Joint (TMJ)

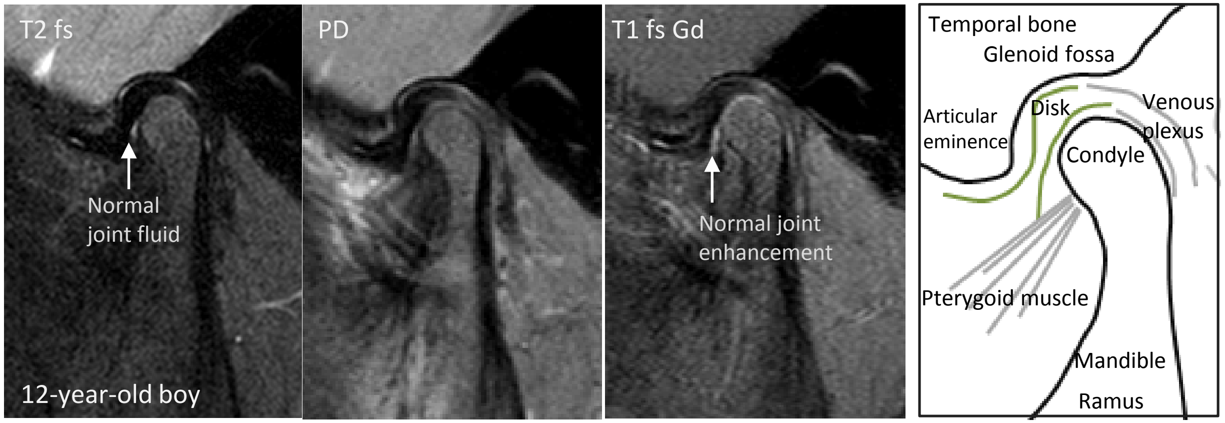

Synovial fluid or joint effusion	Isointense signal compared to cerebrospinal fluid (CSF) on fluid sensitive-images. Small amount of fluid can be seen in normal TMJs.
Synovium	Intermediate signal structure on fluid-sensitive images. Normal synovial lining is not perceptible.
Articular disk	Hypointense intraarticular biconcave structure in all sequences sitting between the head of mandibular condyle and the glenoid fossa of the temporal bone.
Joint enhancement	A normal TMJ can minimally enhance along the joint space, without any evidence of abnormal widening of the joint space and present with signal intensity higher than adjacent musculature and iso- or hypointense to vessels.

Age-Related Changes in Osseous Configuration and Bone Marrow Composition

Shape and contour of mandibular condyle show increased anterior tilt and changes from rounded to oval contour over time

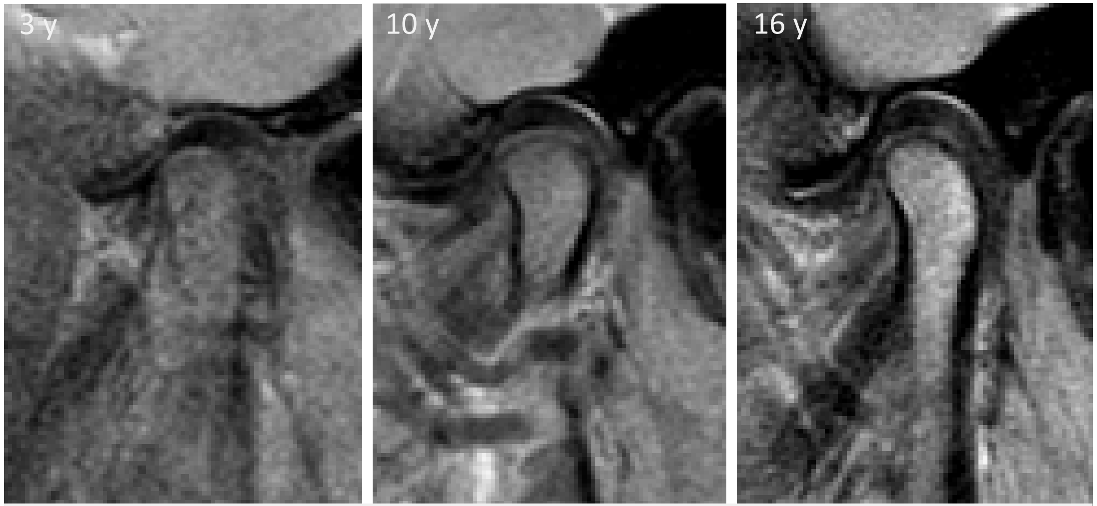

Sagittal-oblique PD-weighted images show configuration of mandibular condyle (left to right): rounded head without a tilt, intermediate appearance and oval head with an anterior tilt

Marrow conversion is expected, changing from hematopoietic to fatty marrow

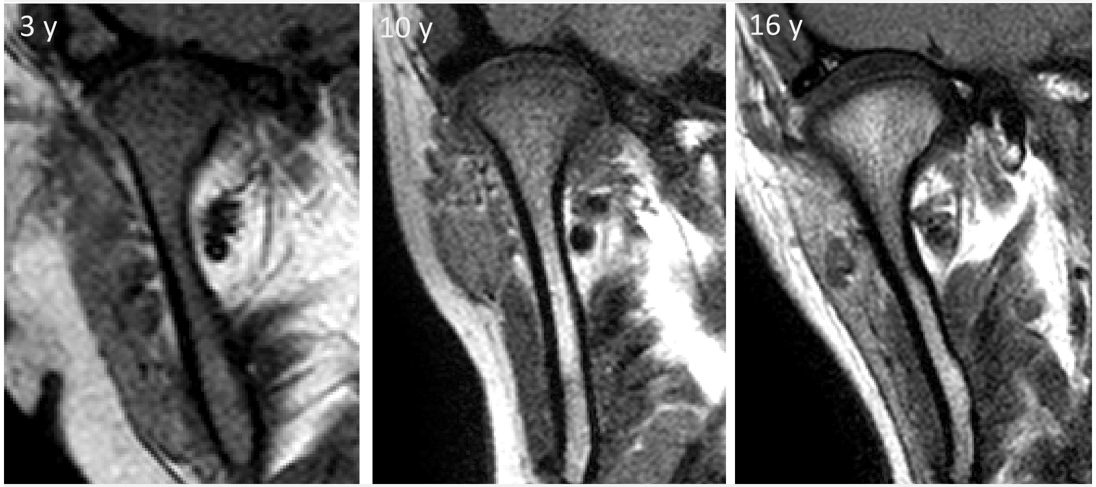

Coronal T1-weighted images show bone marrow type (left to right): hematopoietic marrow, mixed and fatty marrow types

TMJ MRI Scoring System According to OMERACT JIA Group

- Assessment of 2 domains:

Inflammatory domain	- Semi-quantitative assessment of bone marrow oedema and enhancement<br>- Semi-quantitative assessment of joint effusion, joint enhancement and synovial thickening
Damage domain	- Semi-quantitative assessment of condylar flattening, bone erosions and disk abnormalities

A) Inflammatory Domain						
		Bone Marrow Oedema	Bone Marrow Enhancement	Joint Effusion	Synovial Thickening	Joint Enhancement
Definition		Compared to the mandibular ramus, hyperintense marrow signalling within the condyle on T2w fs or STIR images, and/or hypointense signalling on pre-contrast T1w images without fs	Compared to the mandibular ramus, hyperintense marrow signalling within the condyle on post-contrast T1w fs images	Increased joint fluid with isointense signalling of joint space compared to that of cerebrospinal fluid on T2w fs or STIR images	Thickened synovial lining of the joint compartments with intermediate signal on T2w images	Signal intensity of the synovium, capsule, and joint fluid higher than that of muscle on post-contrast T1w fs images
Grading	0	Absent	Absent	Absent: ≤1mm fluid in joint recess	Absent: No synovium visible (joint space ≤1mm width)	Normal: High signal intensity confined to signal perimeter of normal amount of fluid on corresponding fluid-sensitive image
	1	Present	Present	Small: >1 and ≤2mm fluid in recess or involving entire joint compartment	Mild: >1 and ≤2mm thickness at the point of maximum synovial thickening	Mild: High signal intensity focally exceeding signal perimeter of physiologic amount of joint fluid on corresponding fluid-sensitive image
	2			Large: >2mm fluid in recess or involving entire joint compartment	Moderate/Severe: >2mm thickness at the point of maximum synovial thickening	Moderate/Severe: High signal intensity diffusely involving one or both joint compartments

B) Damage Domain				
		Condylar Flattening	Erosions	Disk Abnormalities
Definition		Loss of the round or slightly angular shape of the condylar head, viewed in the sagittal-oblique plane	Any irregularity or break of the bony joint surfaces leading to the loss of the smooth continuous outline of the bone	Any abnormality of the articular disk, including flattening, displacement or destruction
Grading	0	Absent: Round/slightly angular shape	Absent: No irregularities or deep breaks	Absent
	1	Mild: Extent of flattening involves part of the surface of the condyle	Mild: Presence of irregularities involving only part of the articular surface of the condyle	Present
	2	Moderate/Severe: Extent of flattening involves the entire surface of the condyle, or loss of height of the condyle	Moderate/Severe: Presence of deep breaks in the subchondral bone seen in two planes, or irregularities involving the entire articular surface of the condyle	

T2w T2-weighted; *fs* fat saturated; *STIR* short tau inversion recovery; *T1w* T1-weighted

Inflammatory Domain

Bone Marrow Oedema

Definition	Compared to the mandibular ramus, hyperintense marrow signalling within the condyle on fluid-sensitive images, and/or hypointense signalling on pre-contrast T1-weighted images without fat saturation
Grading	Absent
	Present

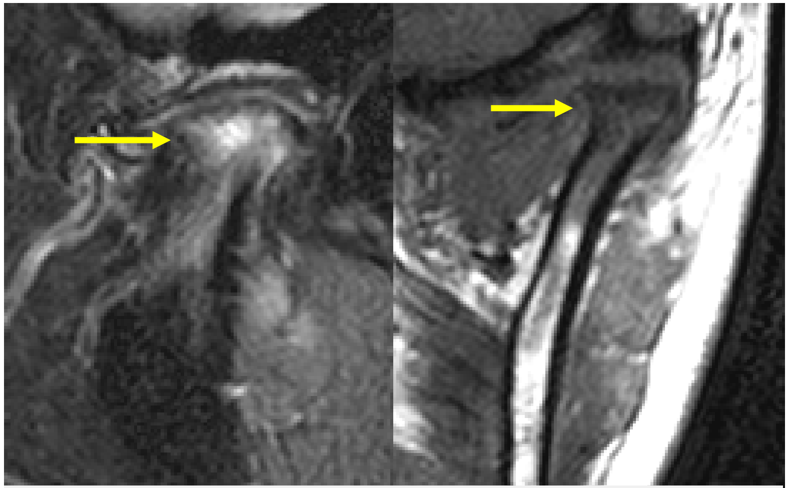

Hyperintense compared to ramus on T2-weighted fat saturated image

Hypointense on pre-contrast T1-weighted image

Inflammatory Domain: Bone Marrow Oedema

Age 0-6 years old

Absent

Sag-obl T2 fs

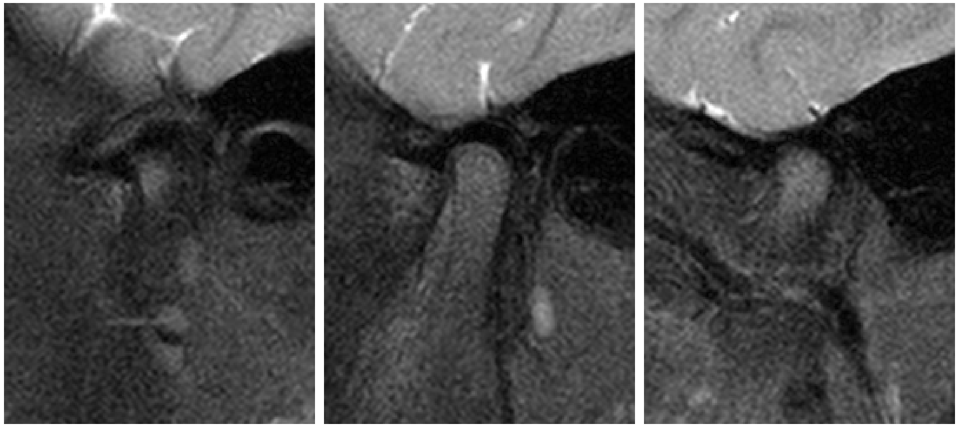

Cor T1

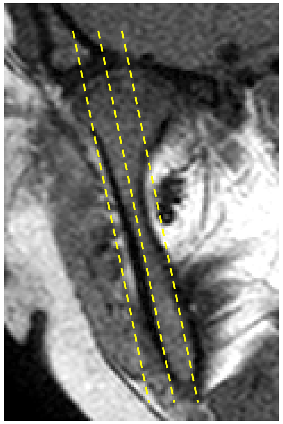

A 3-year-old boy with an unremarkable TMJ

Present

Sag-obl T2 fs

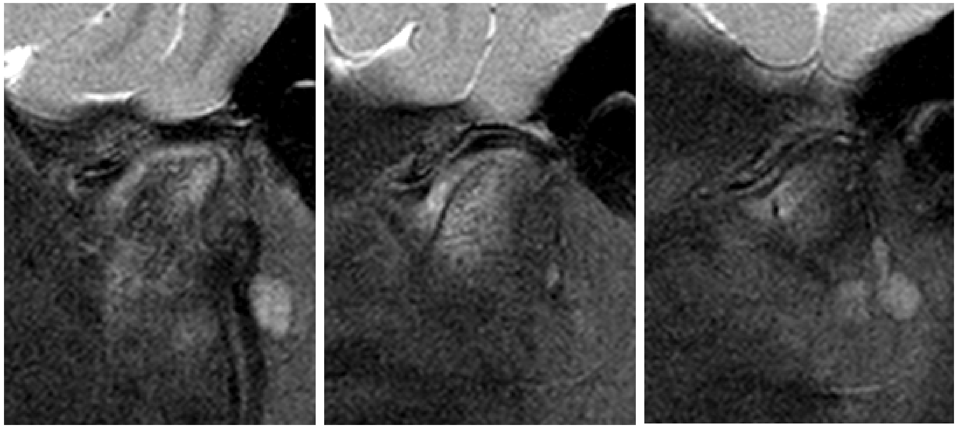

Cor T1

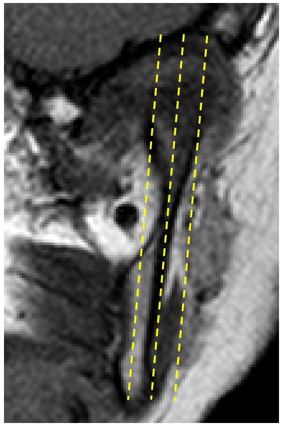

Left TMJ of a 4-year-old boy with a known history of JIA

Age 7-13 years old

Absent

Sag-obl T2 fs

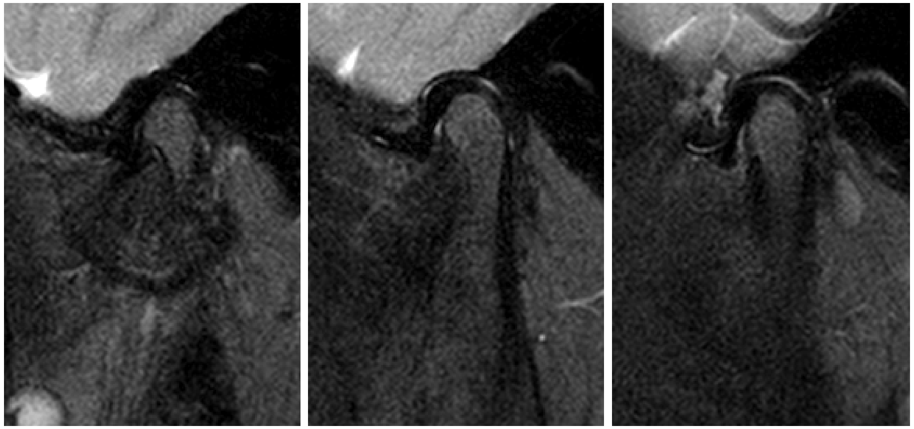

Cor T1

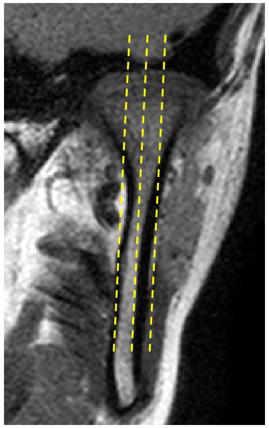

A 12-year-old boy with an unremarkable TMJ

Present

Sag-obl T2 fs

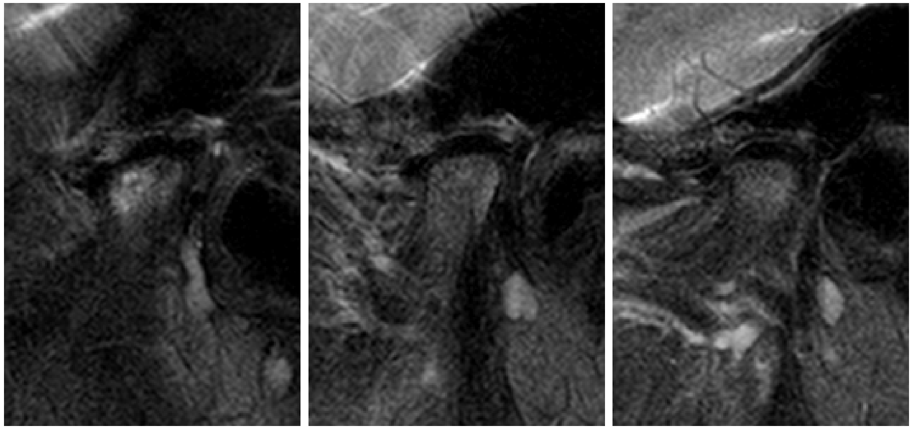

Cor T1

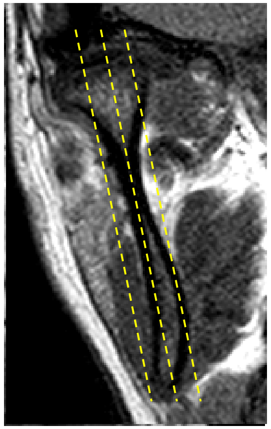

Right TMJ of a 13-year-old girl with a known history of JIA

Age 14-18 years old

Absent

Sag-obl T2 fs

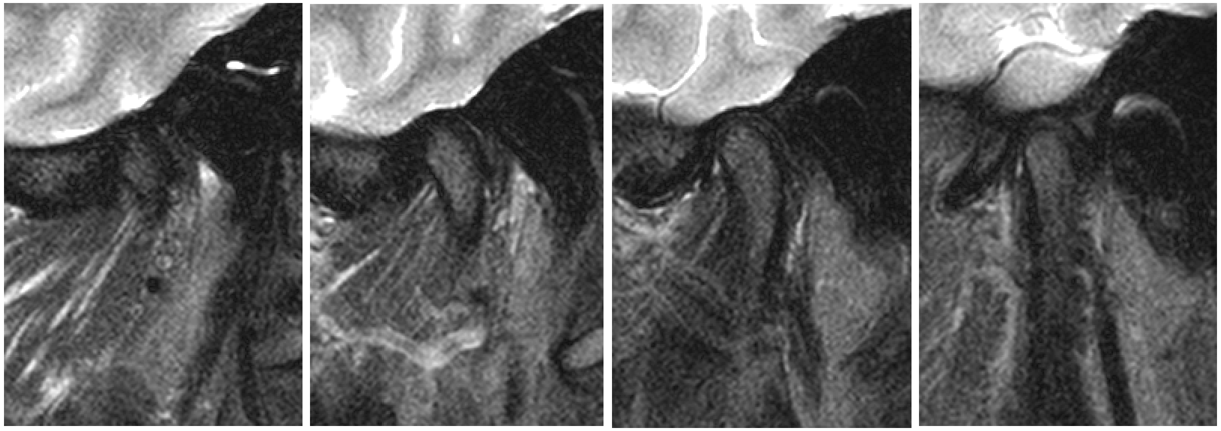

Cor T1

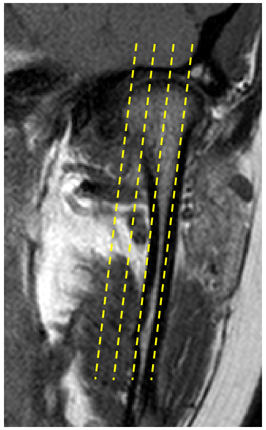

TMJ of a 17-year-old girl with an unremarkable TMJ

Present

Sag-obl T2 fs

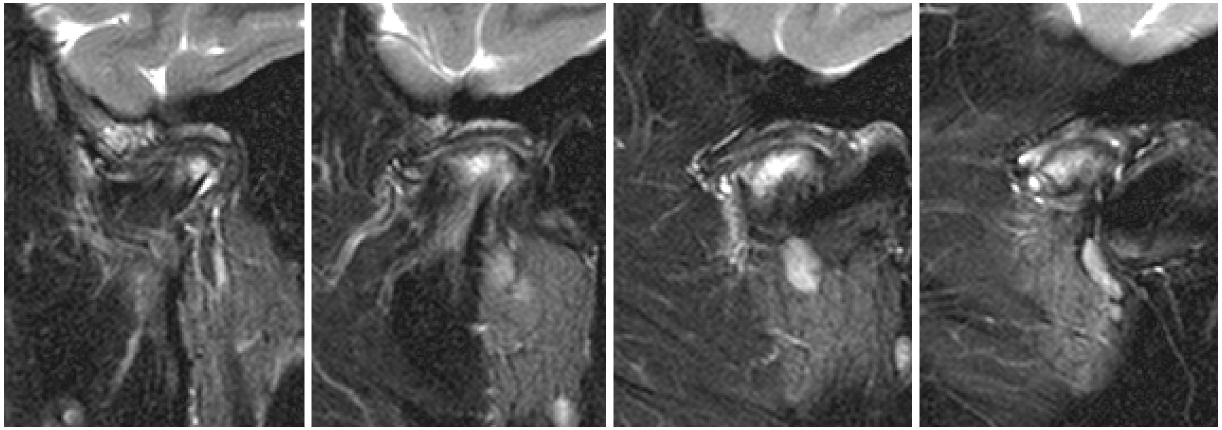

Cor T1

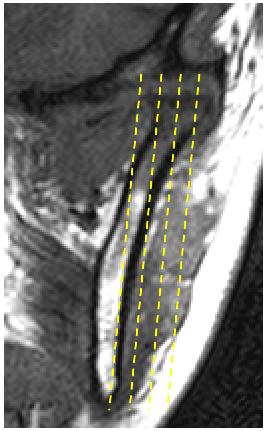

Left TMJ of a 17-year-old girl with a known history of JIA

Inflammatory Domain

Bone Marrow Enhancement

Definition	Compared to the mandibular ramus, hyperintense marrow signalling within the condyle on post-contrast T1-weighted fat-saturated images
Grading	Absent
	Present

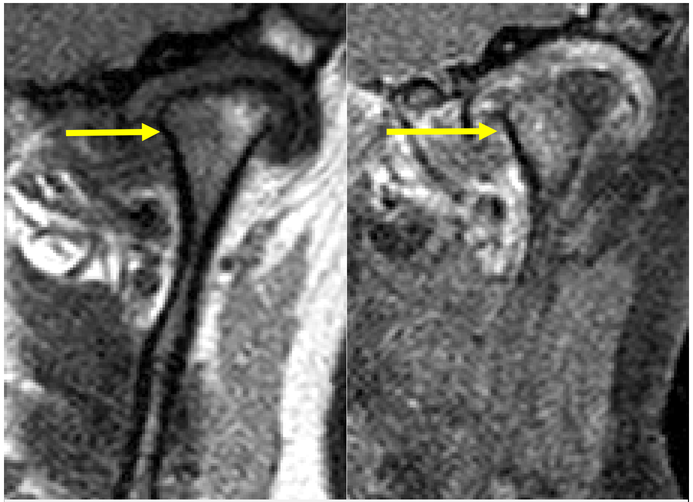

Hypointense signal on pre-contrast T1-weighted image (left) shows higher signal intensity than adjacent marrow on post-contrast T1-weighted fat-saturated image (right)

Inflammatory Domain: Bone Marrow Enhancement

Absent

Cor T1

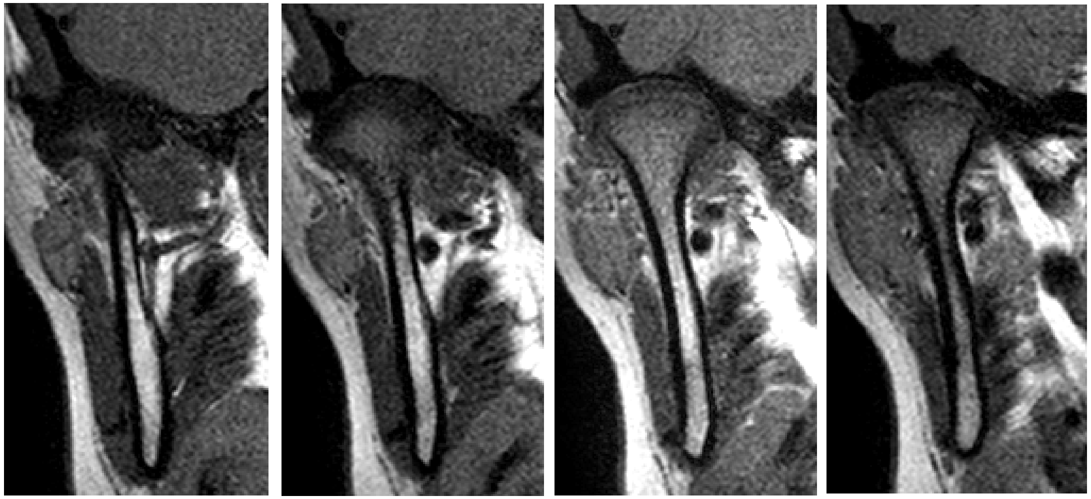

Cor T1 fs Gd

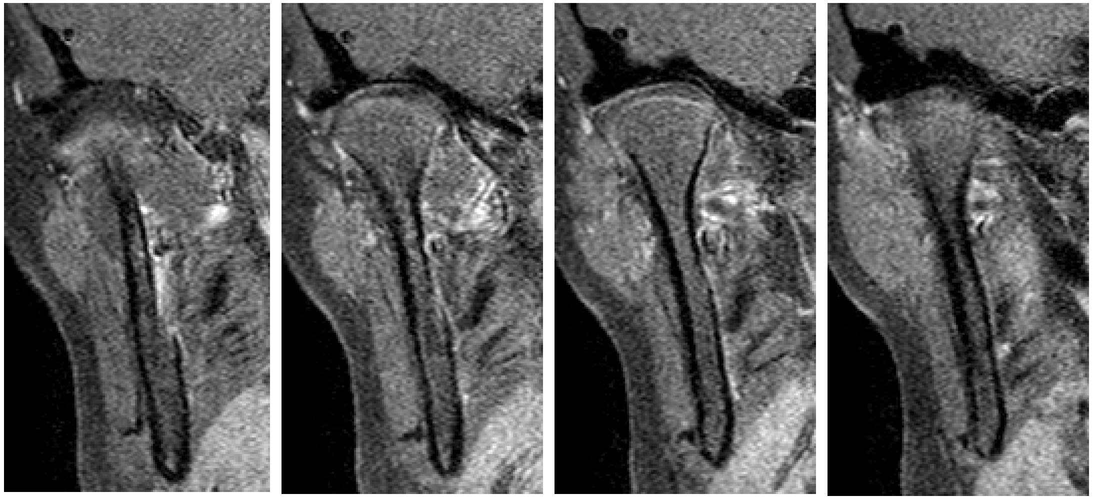

Ax T1 fs Gd

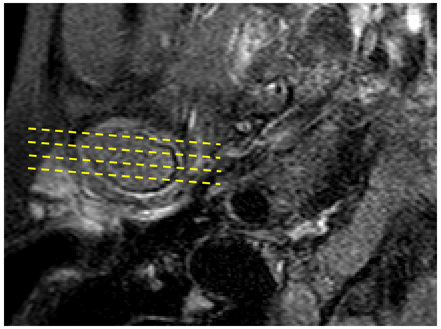

Right TMJ of a 12-year-old boy

Inflammatory Domain: Bone Marrow Enhancement

Present

Cor T1

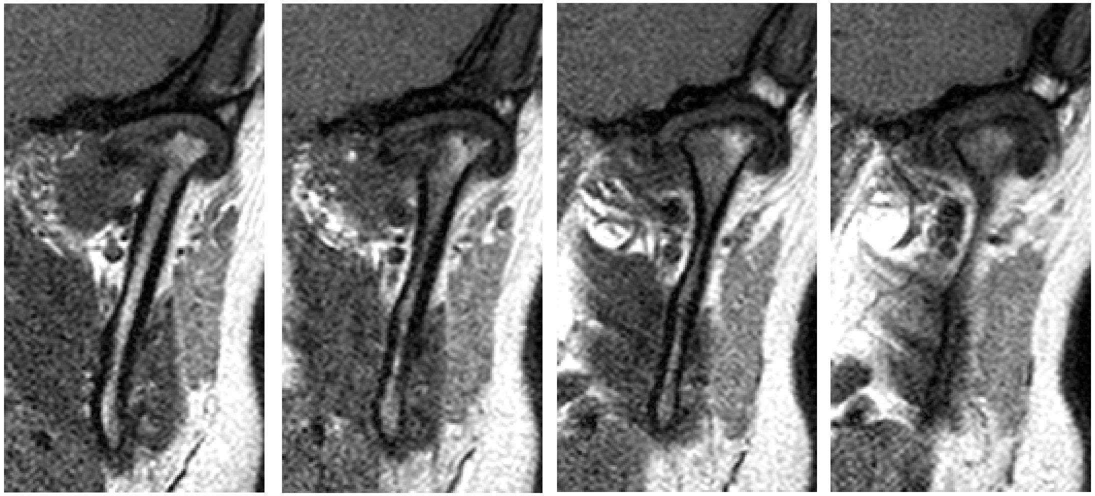

Cor T1 fs Gd

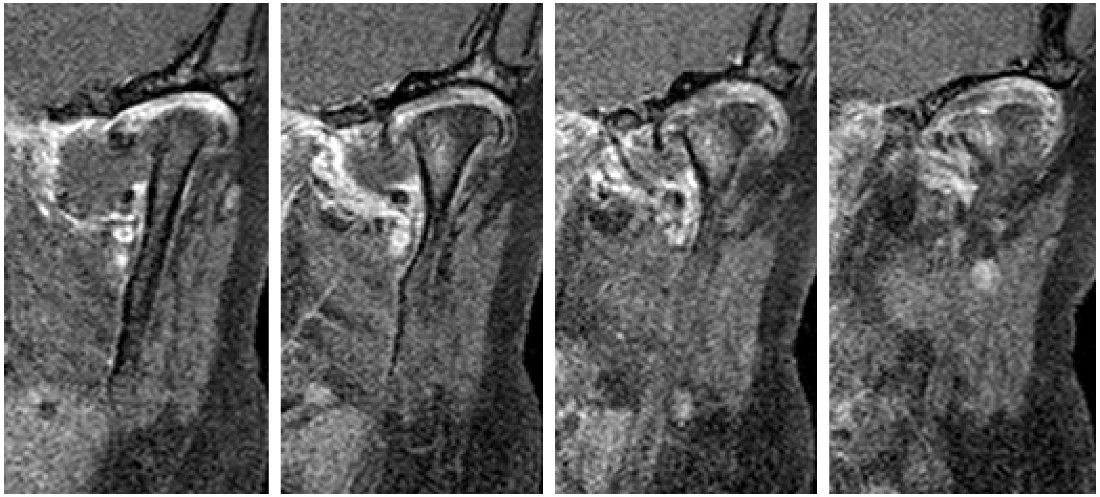

Ax T1 fs Gd

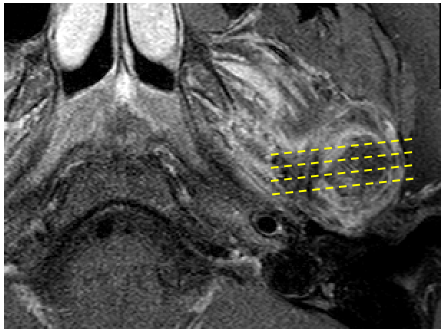

A 12-year-old boy with a known history of JIA

Inflammatory Domain

Joint Effusion

Definition	Increased joint fluid with isointense signalling of joint space compared to that of cerebrospinal fluid on fluid-sensitive images
Grading	Absent:<br>≤1mm fluid in joint recess
	Small:<br>>1 and ≤2mm fluid in recess or involving entire joint compartment
	Large:<br>>2mm fluid in recess or involving entire joint compartment

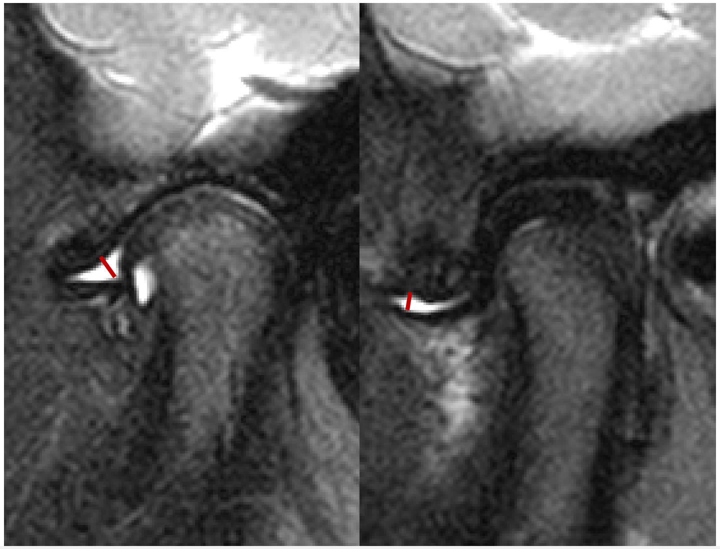

Measuring joint effusion in the largest joint recess on sagittal-oblique T2-weighted fat-saturated image

Inflammatory Domain

Differentiation between Effusion and Synovium

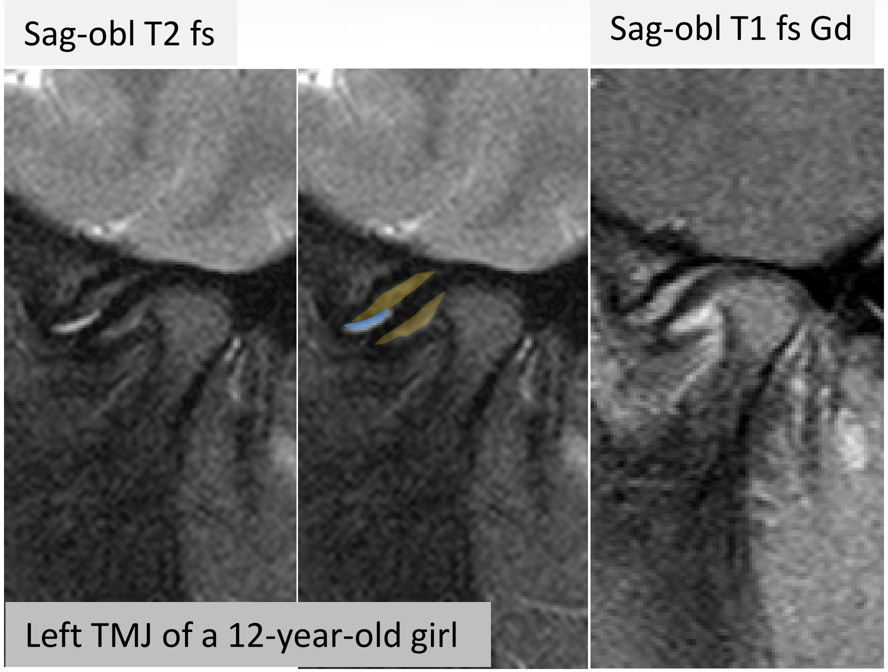

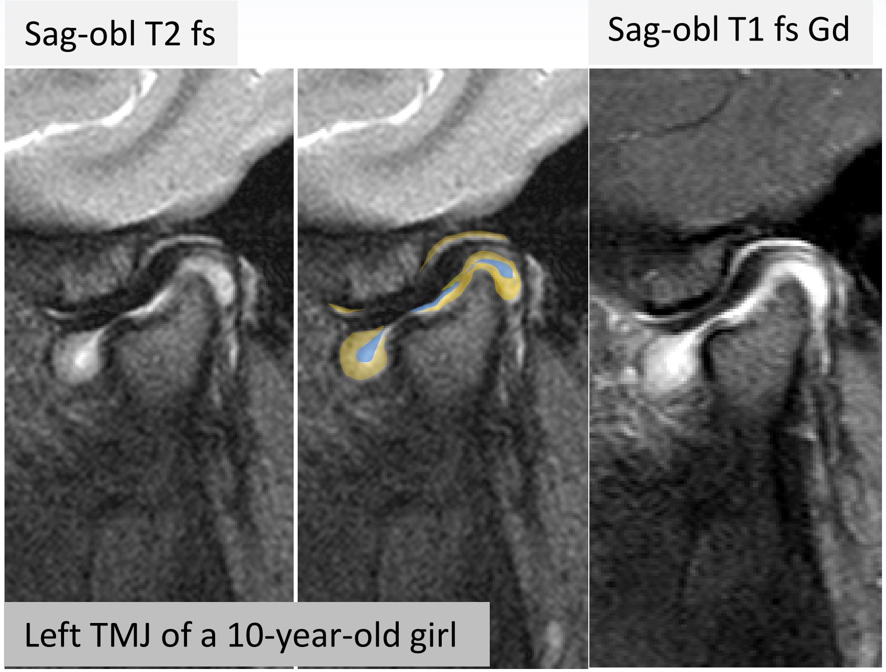

Synovial fluid	Intraarticular isointense signal structure on T2-weighted fat-saturated image compared to signal of CSF, which may enhance after contrast administration
Synovium	Intermediate signal intensity structure on T2-weighted fat-saturated image, which enhances after contrast administration

Inflammatory Domain: Joint Effusion

Sag-obl T2 fs

Cor T1

Absent

Mild

Moderate/ Severe

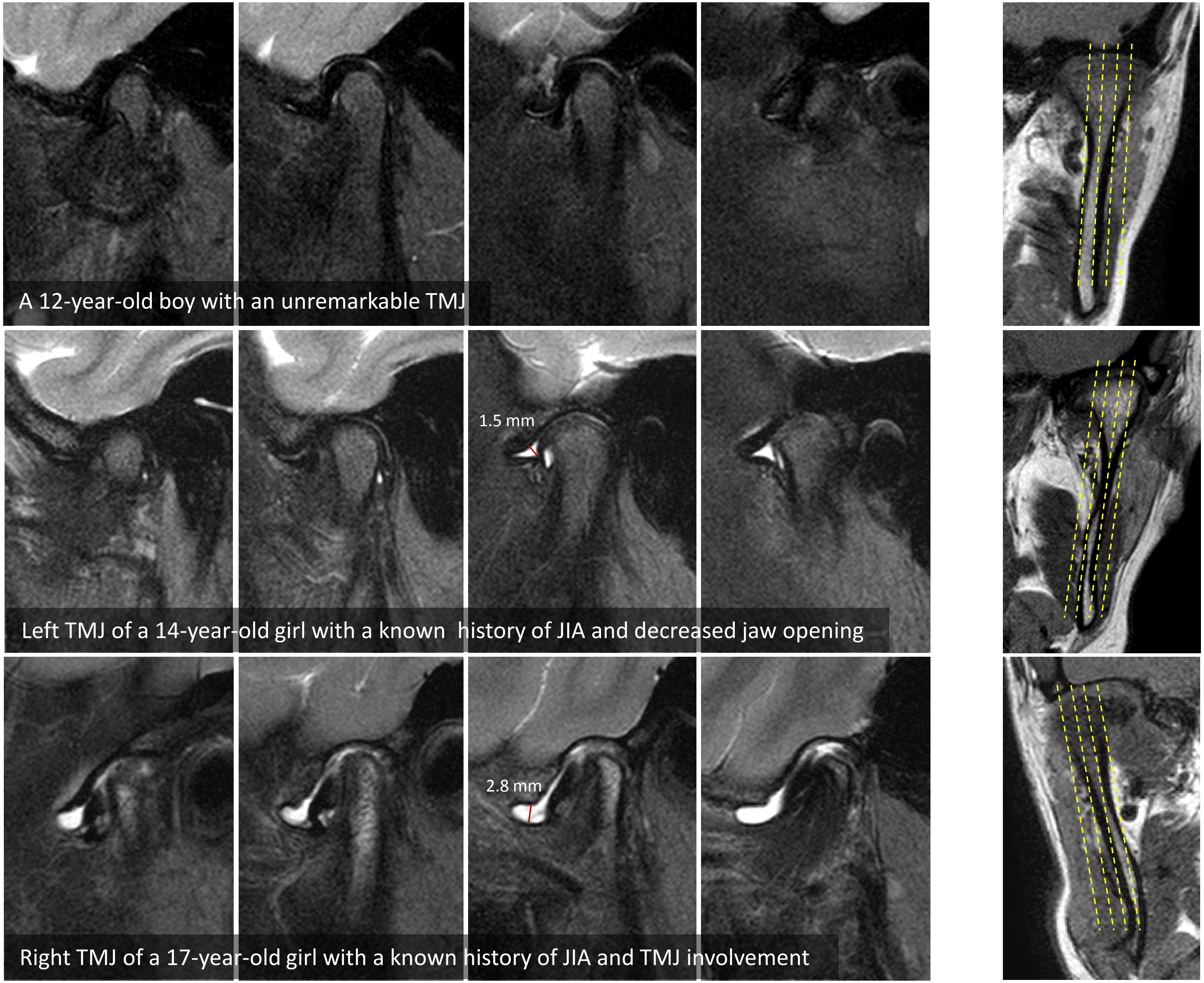

Inflammatory Domain

Joint Enhancement

Definition	Signal intensity of the synovium, capsule, and joint fluid higher than that of muscle on post-contrast T1-weighted fat-saturated images
Grading	Normal:<br>High signal intensity confined to signal perimeter of normal fluid on corresponding fluid-sensitive image
	Mild:<br>High signal intensity exceeding signal perimeter of normal fluid on corresponding fluid-sensitive image
	Moderate/Severe:<br>High signal intensity diffusely involving one or both joint compartments

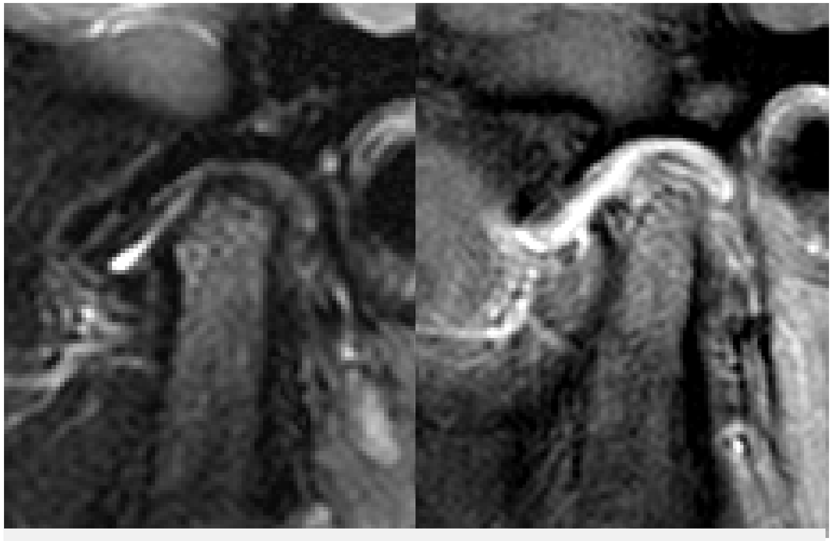

Sagittal T2-weighted fat-saturated (left) and post-contrast T1-weighted fat-saturated (right) images demonstrate joint enhancement

Inflammatory Domain: Joint Enhancement

Normal

Sag-obl T2 fs

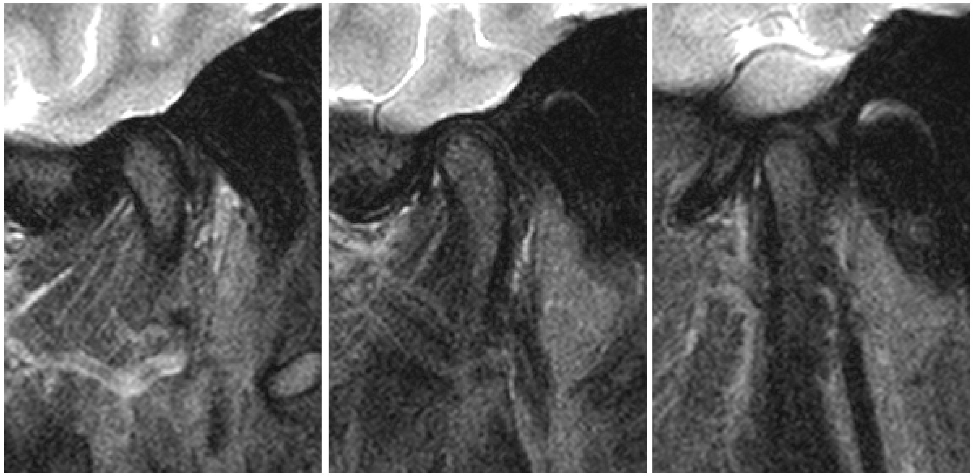

Cor T1

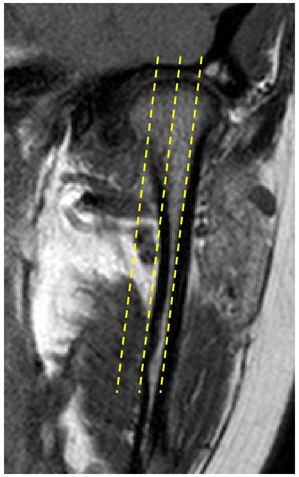

Sag-obl T1 fs Gd

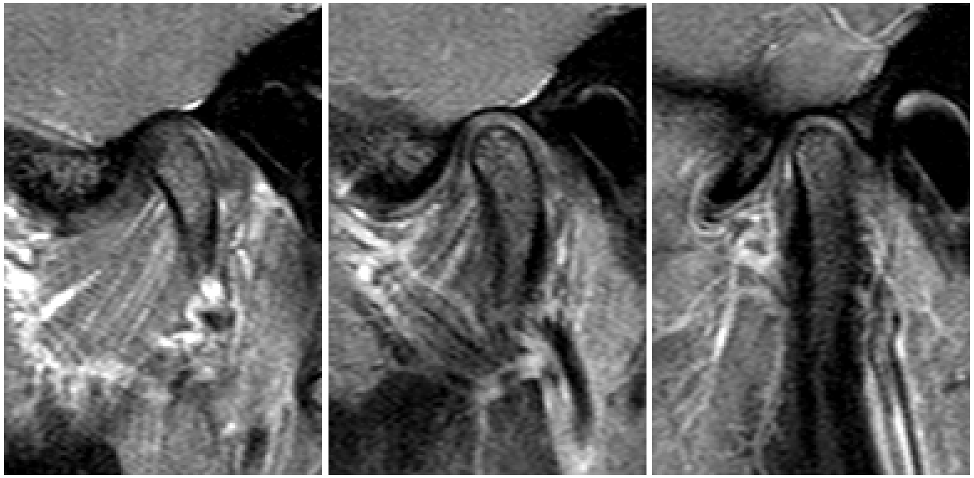

Cor T1 fs Gd

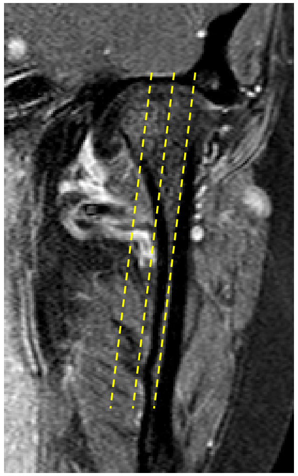

An unremarkable TMJ of a 16-year-old girl presented with knee pain and right jaw click with pain

Mild

Sag-obl T2 fs

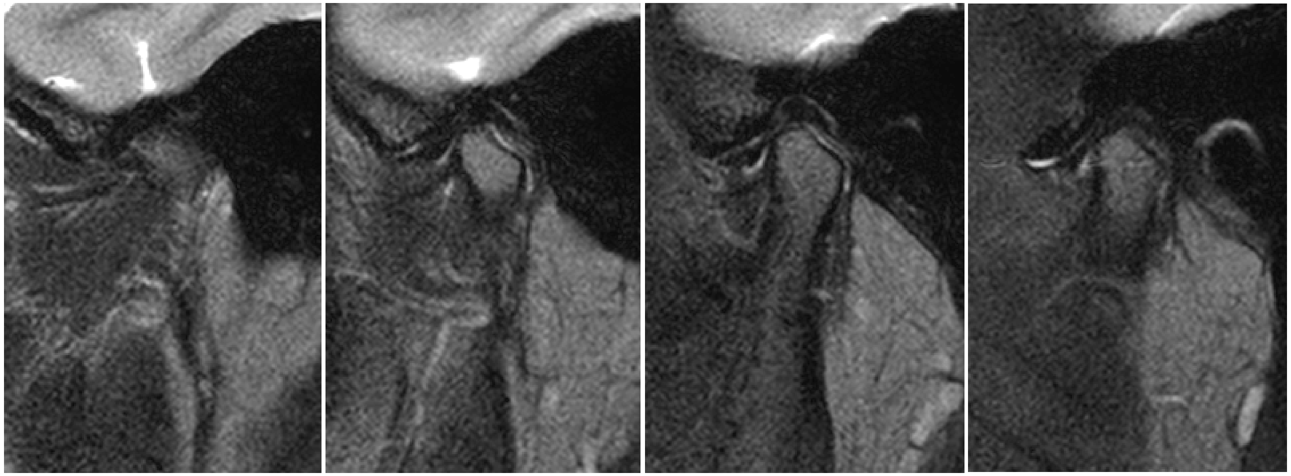

Cor T1

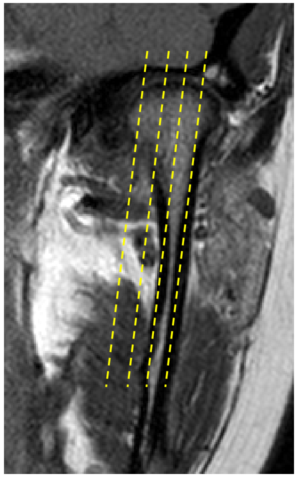

Sag-obl T1 fs Gd

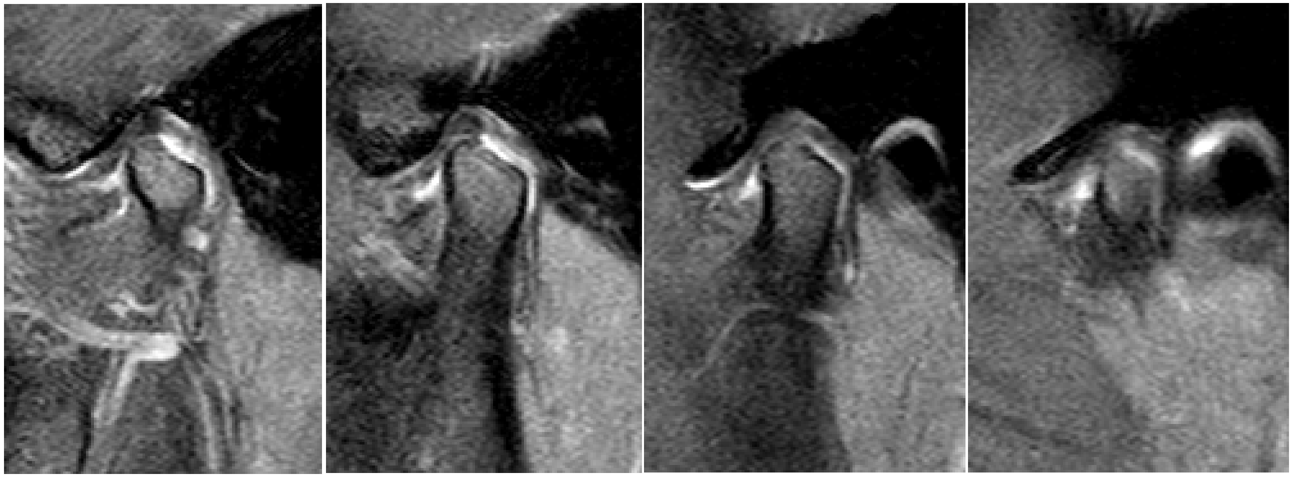

Cor T1 fs Gd

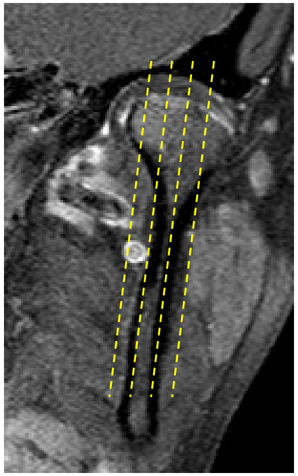

Left TMJ of a 17-year-old girl with a known history of JIA

Inflammatory Domain: Joint Enhancement

Moderate/ Severe

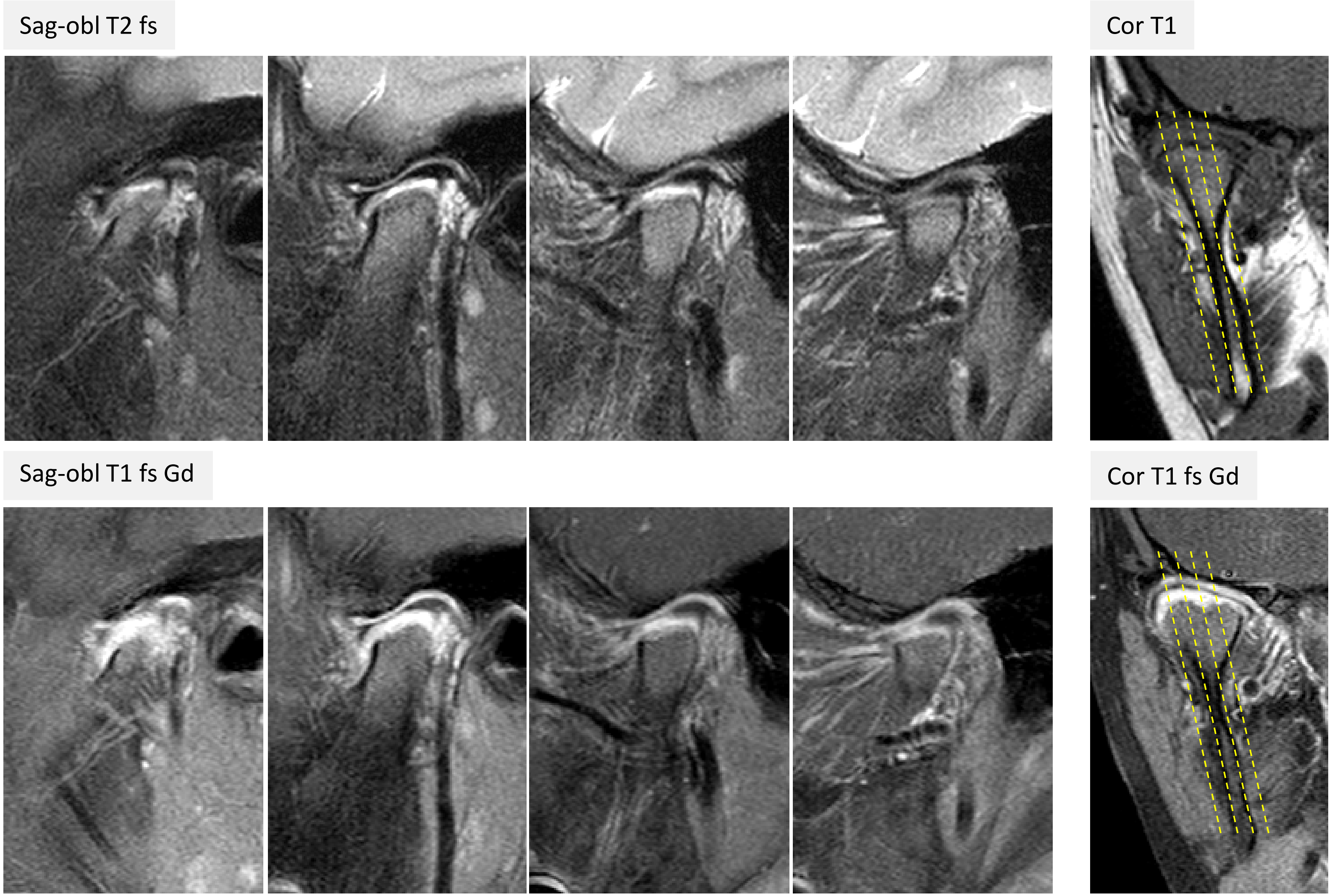

Right TMJ of a 7-year-old boy with a known history of JIA

Inflammatory Domain

Synovial Thickening

Definition	Thickened synovial lining of the joint compartments with intermediate signal intensity on fluid-sensitive images
Grading	Absent:<br>No synovium visible (apparent joint space ≤1mm width)
	Mild:<br>>1 and ≤2 mm thickness at the point of maximum synovial thickening
	Moderate/Severe:<br>>2mm thickness at the point of maximum synovial thickening

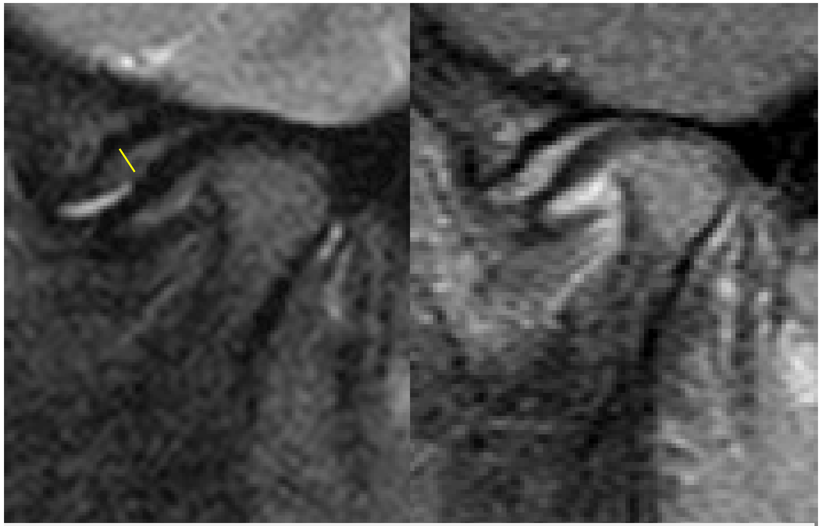

Maximal thickness of synovium is measured on sag-obl T2 fs (left) which enhances on sag-obl T1 fs Gd (right)

Inflammatory Domain: Synovial Thickening

Absent

Sag-obl T2 fs

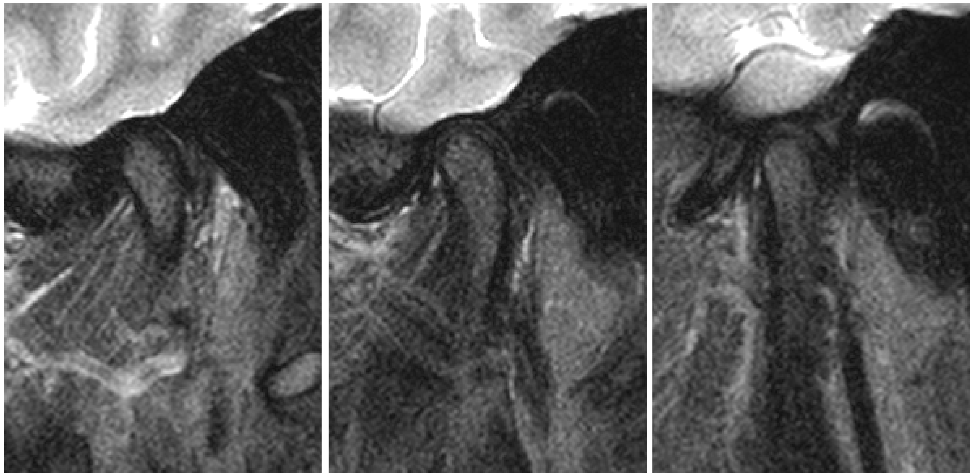

Cor T1

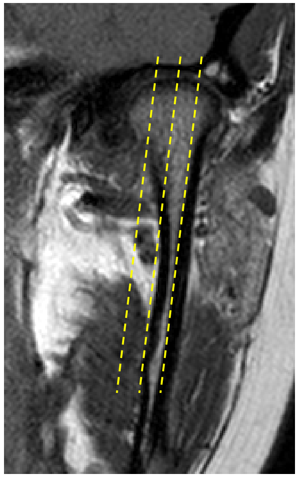

Sag-obl T1 fs Gd

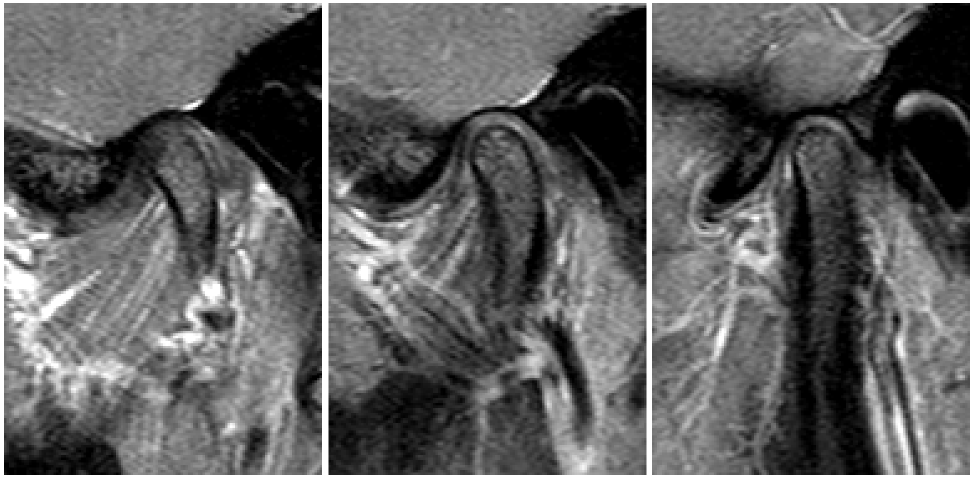

Cor T1 fs Gd

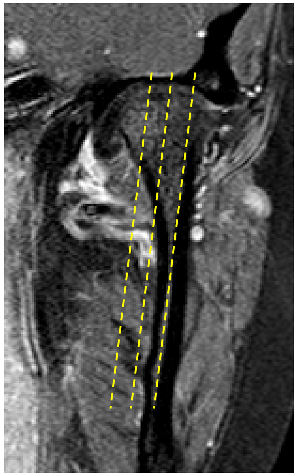

An unremarkable TMJ of a 16-year-old girl who presented with knee pain and right jaw click with pain

Inflammatory Domain: Synovial Thickening

Mild

Sag-obl T2 fs

Cor T1

Sag-obl T1 fs Gd

Cor T1 fs Gd

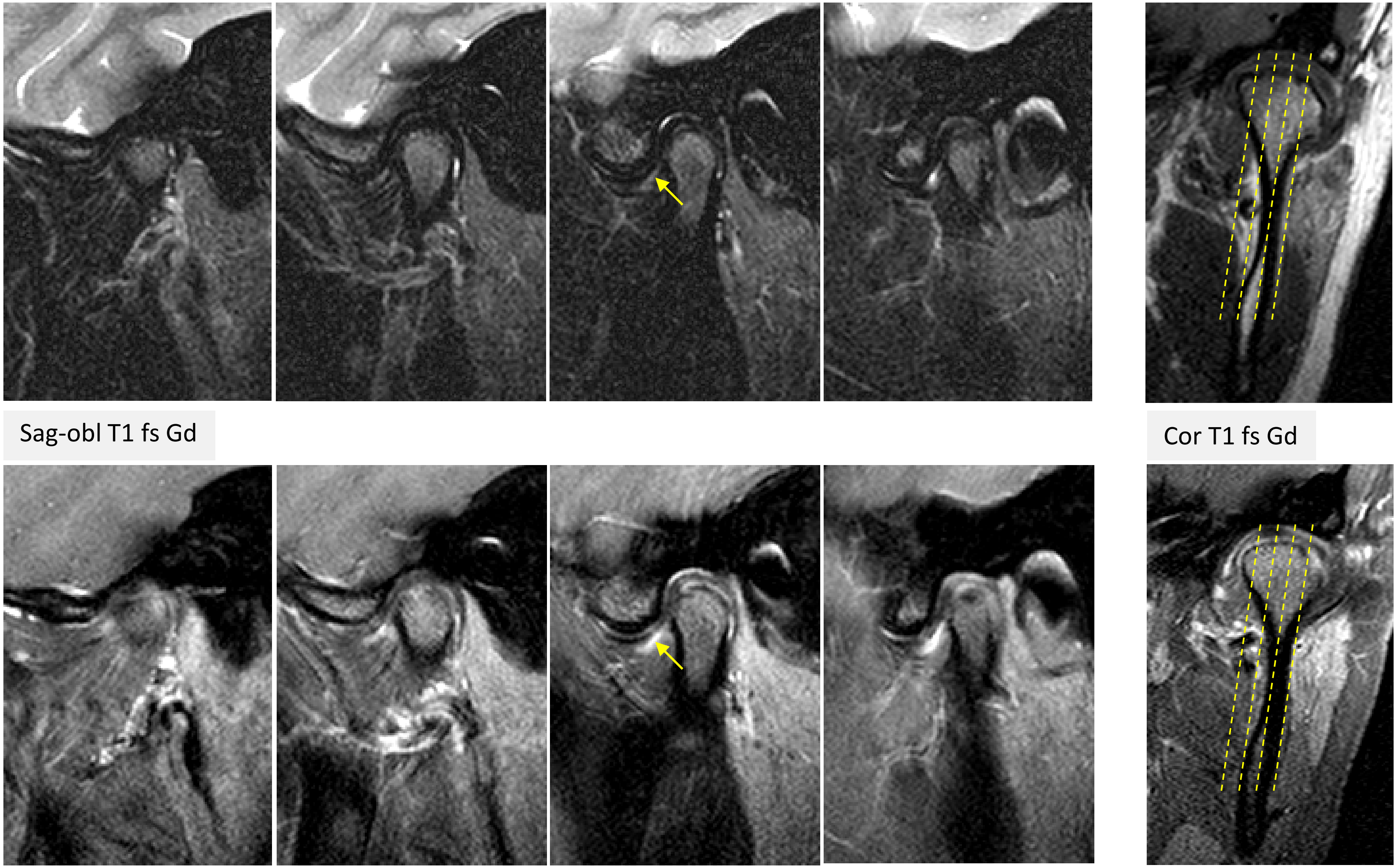

Left TMJ of a 16-year-old girl with a known history of extended oligoarticular JIA and TMJ involvement

Moderate/ Severe

Sag-obl T2 fs

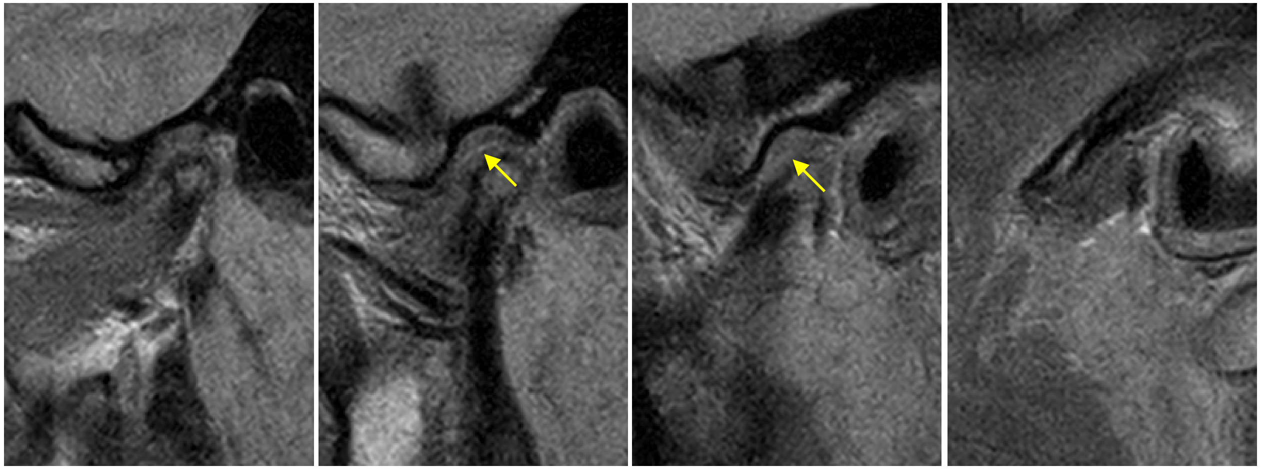

Cor T1

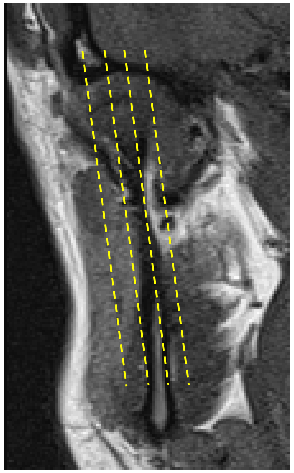

Sag-obl T1 fs Gd

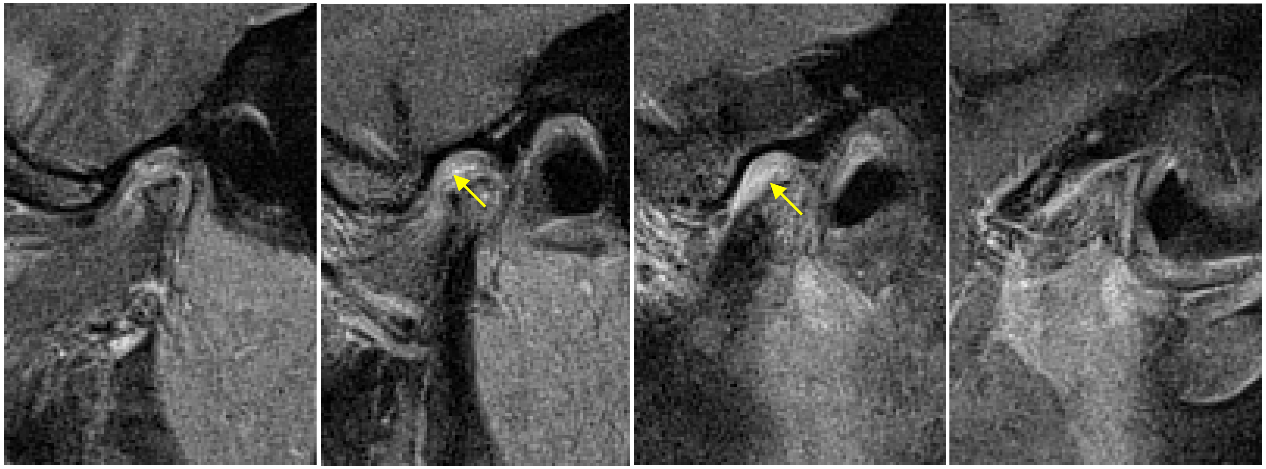

Cor T1 fs Gd

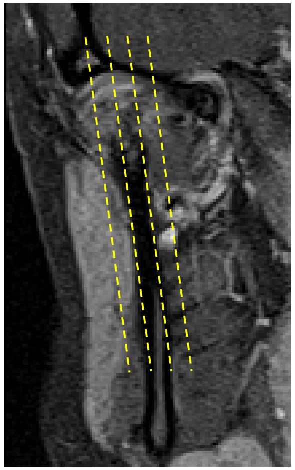

A 16-year-old girl with a known history of oligoarticular JIA with right TMJ arthritis

Damage Domain

Condylar Flattening

Definition	Loss of the round or slightly angular shape of the condylar head, viewed in the sagittal-oblique plane
Grading	Absent:<br>Normal round/slightly angular shape
	Mild:<br>Extent of flattening involves part of the surface of the condyle
	Moderate/Severe:<br>Extent of flattening involves the entire surface of the condyle, or loss of height of the condyle

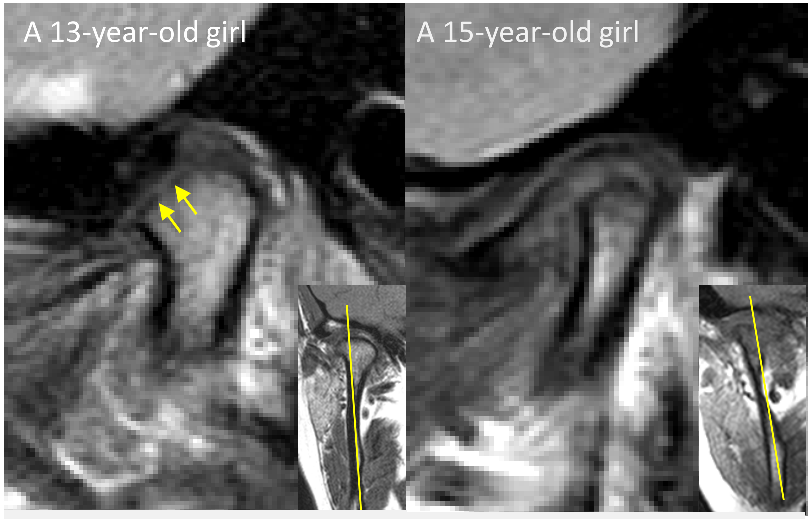

Sag-obl PD image shows loss of the rounded shaped of the condyle

Sag-obl PD image shows hypoplastic condyle with flattened anterior surface

Age 0-6 years old

Damage Domain: Condylar Flattening

Sag-obl PD

Cor T1

Absent

Mild

Moderate/ Severe

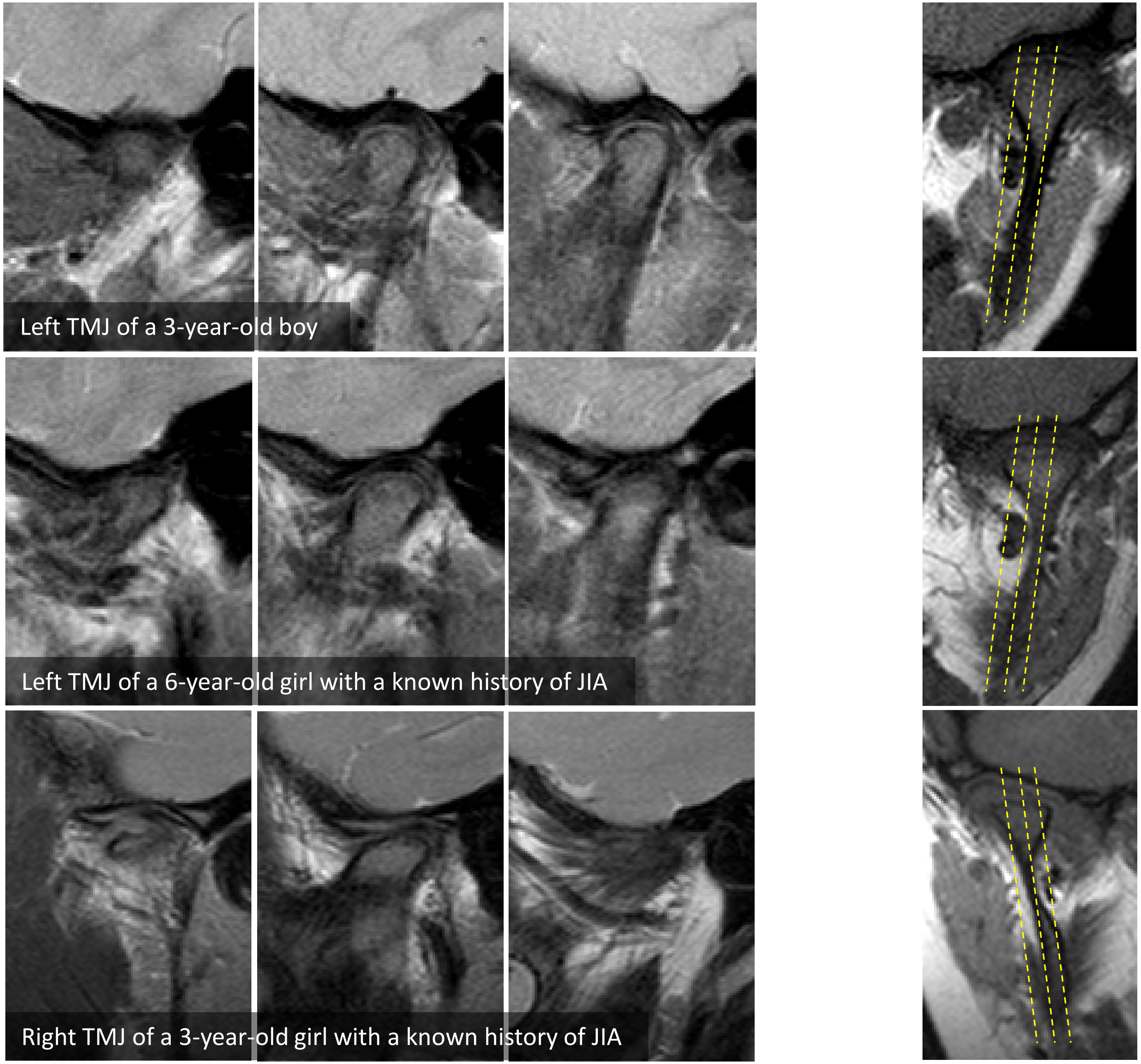

Damage Domain: Condylar Flattening

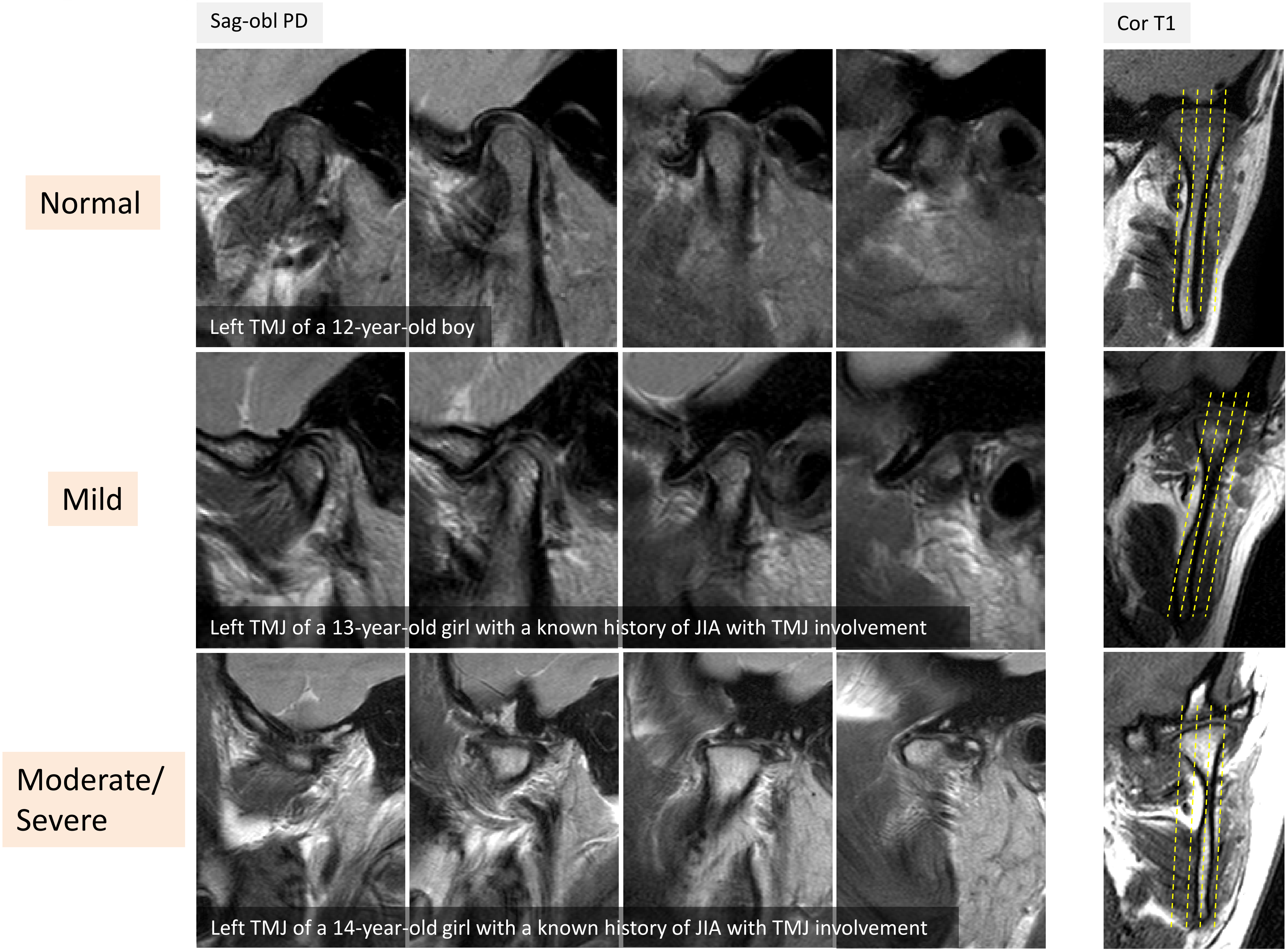

Age 15-18 years old

Damage Domain: Condylar Flattening

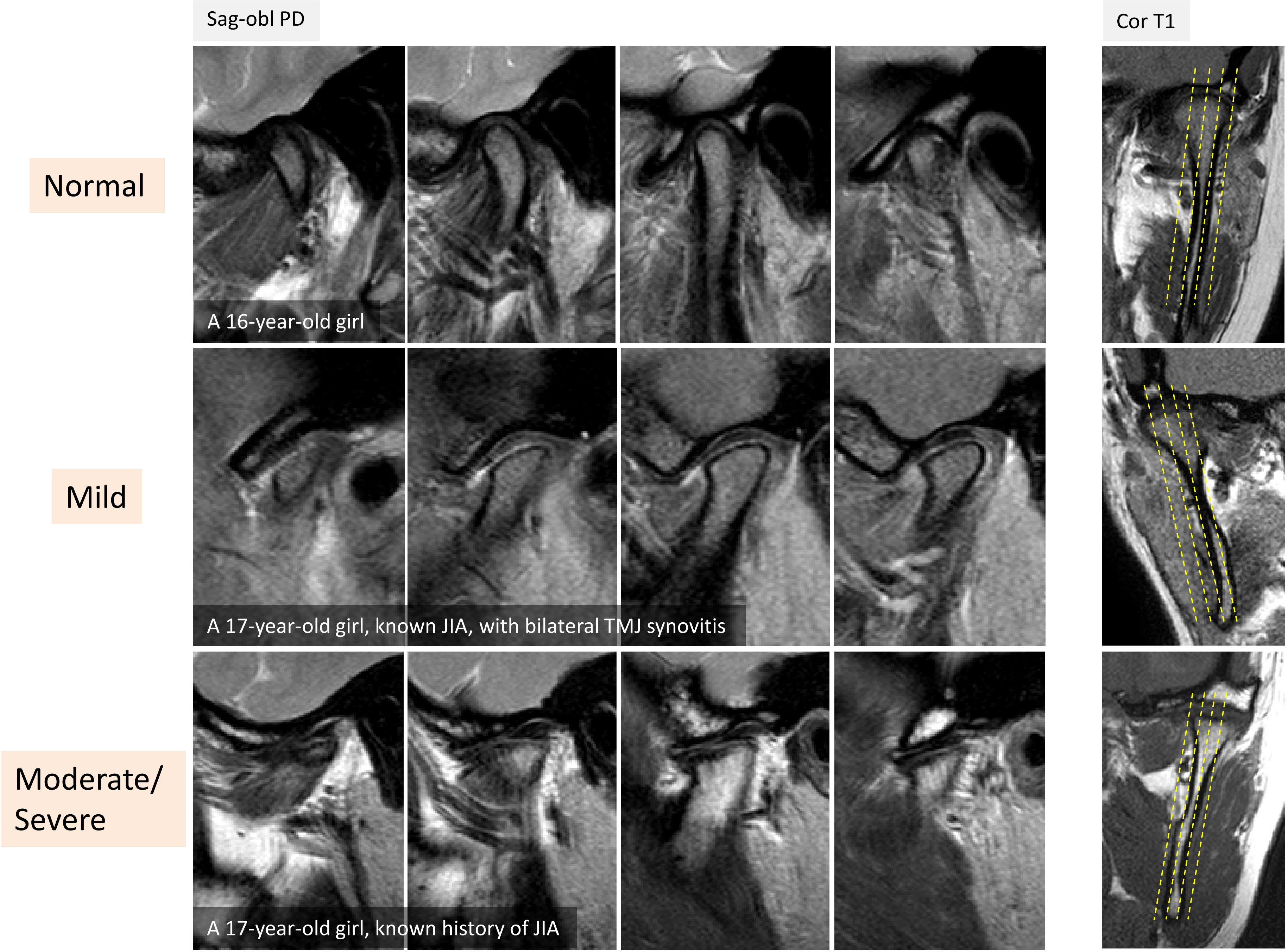

Damage Domain

Erosions

Definition	Any irregularity or break of the bony joint surfaces leading to the loss of the smooth continuous outline of the bone
Grading	Absent: No irregularities or deep breaks
	Mild: Presence of irregularities involving only part of the articular surface of the condyle
	Moderate/Severe: Presence of deep breaks in the subchondral bone seen in two planes, or irregularities involving the entire articular surface of the condyle

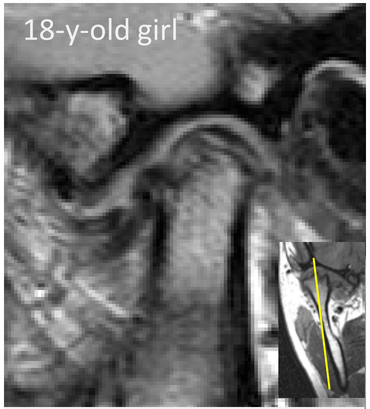

Sagittal PD-weighted image shows breaks of joint surface

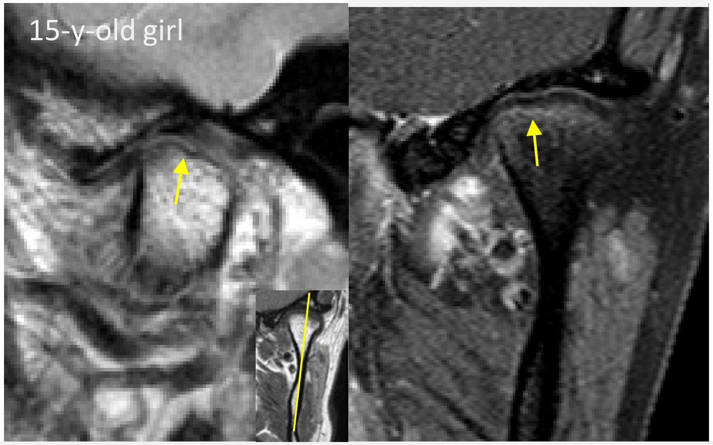

Sagittal PD-weighted (left) and post-contrast coronal T1-weighted fat-saturated (right) images show surface irregularity

Damage Domain: Erosions

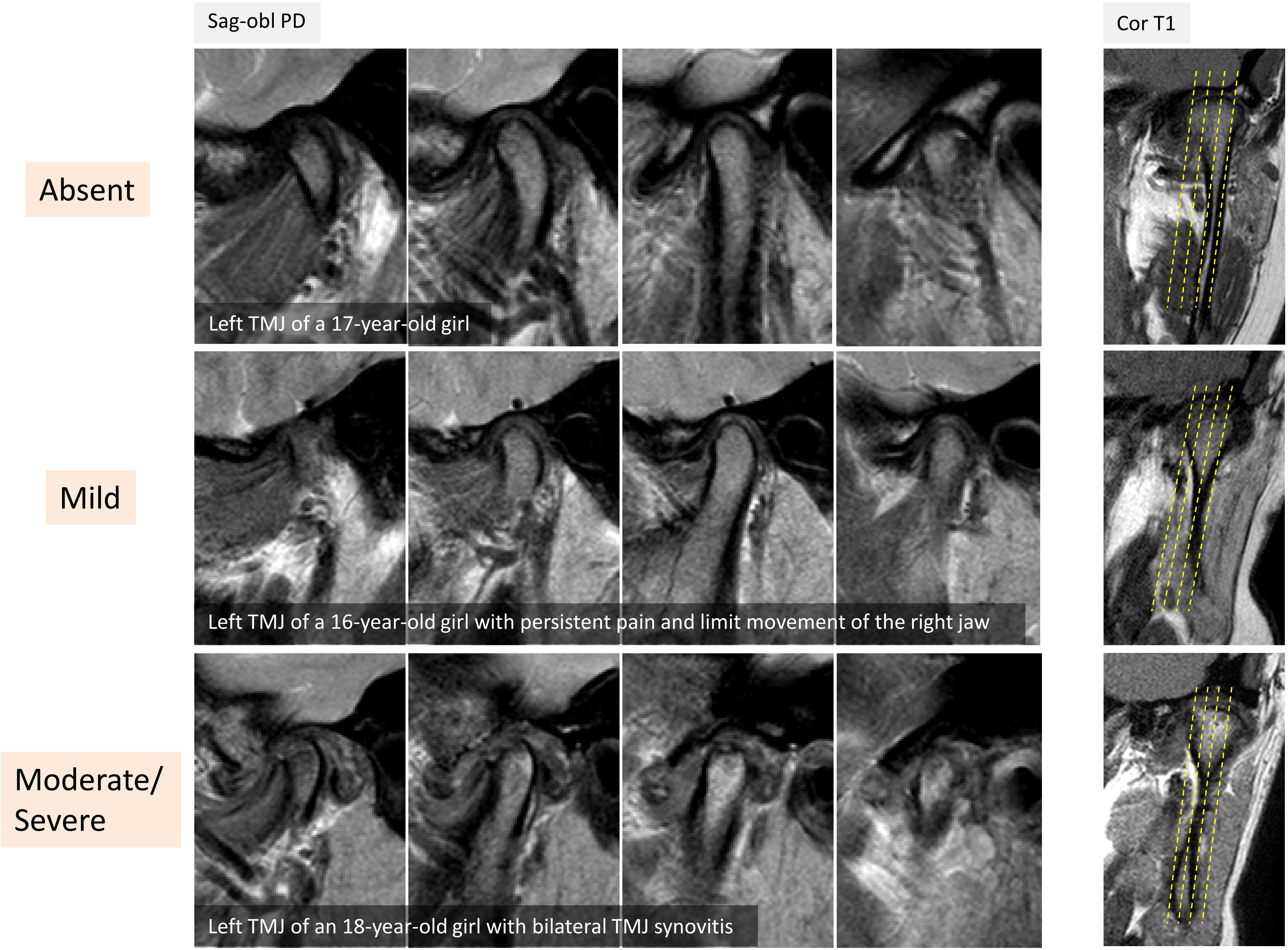

Damage Domain

Disk Abnormalities

Definition	Any abnormality of the articular disk, including flattening, displacement or destruction
Grading	Absent
	Present

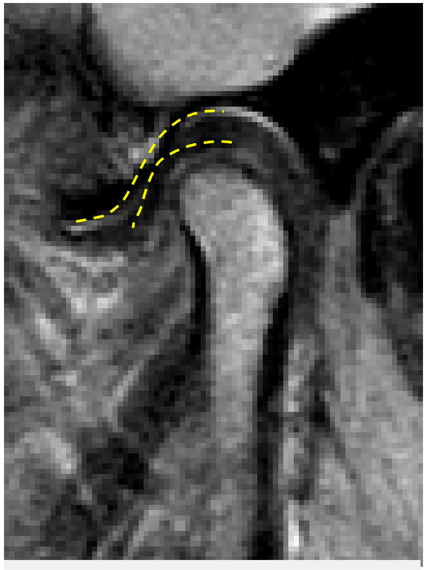

Sagittal-oblique PD-weighted image shows normal intraarticular disk

Damage Domain: Disk Abnormalities

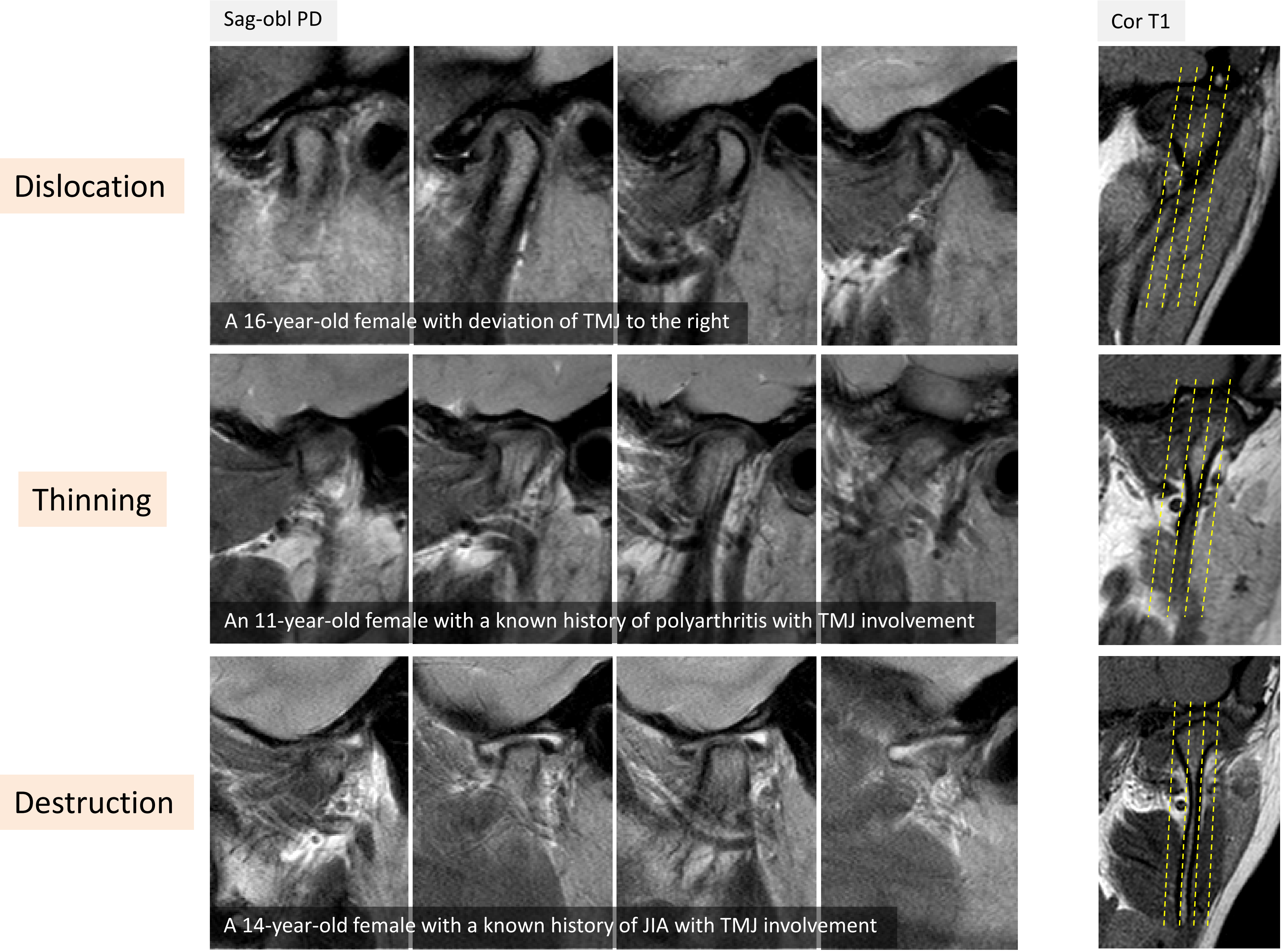

Abbreviations in the Imaging Atlas

- Abbreviations:

CSF	Cerebrospinal Fluid
JIA	Juvenile Idiopathic Arthritis
MRI	Magnetic Resonance Imaging
OMERACT	Outcome Measures in Rheumatic Arthritis and Clinical Trials
TMJ	Temporomandibular Joint
y	years

- MRI sequences:

T1	T1-weighted
T2	T2-weighted
PD	Proton density weighted
fs	fat-saturated
Gd	Gadolinium enhanced

- Imaging planes:

Sag-obl	Sagittal-oblique
Cor	Coronal
Cor-obl	Coronal-oblique

SickKids

UNIVERSITY
CHILDREN'S HOSPITAL
ZURICH
