# Supplementary material for: Temporomandibular joint atlas for detection and grading of juvenile idiopathic arthritis involvement by magnetic resonance imaging
Source: Pediatr Radiol. 2017 Nov 13;48(3):411–26. doi: 10.1007/s00247-017-4000-0 (PMC5823950; doi:10.1007/s00247-017-4000-0)
Supplement: Supplementary file 2 — (PDF 96 kb) [file 247_2017_4000_MOESM2_ESM.pdf]

Imaging parameters for temporomandibular joint MRI

Online Resource 2 to pictorial essay “Temporomandibular Joint Atlas for Detection and Grading of Juvenile Idiopathic Arthritis Involvement by Magnetic Resonance Imaging”, Pediatric Radiology.

Christian J. Kellenberger, Thitiporn Junhasavasdikul, Mirkamal Tolend, Andrea S. Doria
Corresponding Author: Christian J. Kellenberger, Department of Diagnostic Imaging, University Children’s Hospital Zürich, christian.kellenberger@kispi.uzh.ch

Protocol from the University Children’s Hospital Zürich for 1.5 Tesla (Discovery MR450, GE Healthcare), obtained with dual ring TMJ coil

	Pre-contrast		Post-contrast			Bone evaluation
Image weighting	PD-weighted	T1-weighted	T2-weighted with fat saturation	T1-weighted with fat saturation	T1-weighted with fat saturation	PD-weighted
Imaging plane	Sagittal-oblique	Sagittal-oblique	Sagittal-oblique	Sagittal-oblique	Coronal	Sagittal-oblique
MRI sequence	FSE	2D FSPGR	FSE	FSE	FSE	3D FSPGR
Flip angle	90°	80°	90°	90°	90°	20°
Repetition time [ms]	3000	300	5400	670	500	10
Echo time [ms]	13	4.2	77	10	10	4.2
Echo train length	8	na	16	4	4	na
Field of view [mm]	120	120	120	120	220	100
Slice thickness / interslice distance [mm]	2/0	2/0	2/0	2/0	3/0	2/-1
Matrix	256 x 224	384 x 224	256 x 224	256 x 192	256 x 192	256 x 192
Signal averages [n]	3	3	3	3	3	3
Images [n]	12 for each TMJ	12 for each TMJ	12 for each TMJ	12 for each TMJ	12	28 for each TMJ

2D two dimensional, *3D* three dimensional, *FSE* fast spin echo, *FSPGR* fast spoiled gradient echo, *na* not applicable, *PD* proton density, *TMJ* temporomandibular joint

Protocol from the Hospital for Sick Children Toronto for 3 Tesla (Philips Achieva), obtained with 32-channel head coil

	Pre-contrast		Post-contrast			
Sequences	T1 TSE	T2 TSE FS	PD TSE	T1 TSE FS	T1 TSE FS	T1 TSE FS
Plane	Coronal	Sagittal-oblique	Sagittal-oblique	Sagittal-oblique	Coronal	Axial
TE [ms]	21	80	30	20	20	20
TR [ms]	600	4000	2000	600	600	600
Flip angle [°]	90	90	90	90	90	90
FOV (mm x mm)	140 x 140	110 x 110	110 x 110	110 x 110	140 x 140	140 x 140
Acquisition matrix	280 x 280	288 x 280	276 x 241	276 x 219	348 x 279	280 x 230
Pixel spacing	0.292 x 0.292	0.196 x 0.196	0.344 x 0.344	0.286 x 0.286	0.312 x 0.312	0.312 x 0.312
Slice thickness (mm)	2	3	3	3	2	3
# of averages	2	3	2	1	1	1
Slice spacing (mm)	2.2	3.3	3.3	3.3	2.2	2.2
Echo train length	4	15	7	3	3	3
Pixel bandwidth	291	210	282	291	291	291

T1 T1-weighted, *T2* T2-weighted, *PD* proton density, *TSE* turbo spin echo, *TE* echo time, *TR* repetition time, *FOV* field of view
